# Mutations in the TP53, VEGFA, and CTH Genes as Key Molecular Markers for the Diagnosis of Glioblastoma

**DOI:** 10.7759/cureus.61165

**Published:** 2024-05-27

**Authors:** Sardar S Khalil, Abbas Salihi

**Affiliations:** 1 Department of Biology, Faculty of Science, University of Zakho, Duhok, IRQ; 2 Department of Biology, College of Science, Salahaddin University-Erbil, Erbil, IRQ

**Keywords:** gene mutation, cth, vegfa, tp53, glioblastoma

## Abstract

Background

Brain cancer, particularly glioblastoma (GBM), is a global health problem. Despite therapy advances, GBM patients have a poor prognosis. The progression and etiology of GBM may be linked to gene polymorphisms in the *VEGFA*, *TP53*, and *CTH* genes, among others. However, the genetic variations and their interaction in GBM are not fully understood. This study examines the effects of mutations in the *VEGFA*, *TP53*, and *CTH* genes on GBM.

Methodology

Tissue and blood samples were obtained for hematological, biochemical, and genetic analysis from 18 patients diagnosed with GBM as well as 28 healthy individuals. Standard methods were adopted to perform hematological and biochemical analyses, whereas mutational landscape and expression profiles were obtained from publicly accessible databases. Tissue samples were processed for genomic DNA extraction, and genotype determination was carried out through conventional polymerase chain reaction (PCR) and Sanger sequencing.

Results

The study involved 18 patients with grade IV GBM before treatment and 28 healthy individuals. The patients consisted of 11 men (61%) and seven females (39%), while healthy individuals included 14 (50%) males and 14 (50%) females. Sixty-seven percent of patients were under 50, 17% between 51 and 60, and 17% over 61, compared to healthy individuals who were 61% under 50, 7% between 51 and 60, and 32% over 60. GBM patients showed higher neutrophil and monocyte counts (median 81% (63.9, 83.5) and 4.2% (3.8-7.3)), respectively, and lower lymphocyte counts (median 13.4% (8.8, 28.40)) compared to controls. The median values of aspartate transaminase (AST), alanine transaminase (ALT), and alkaline phosphatase (ALP) showed no significant differences between the control and GBM groups. GBM patients had significantly higher median CRP levels of 2.55 (1.6, 98) than controls. Analysis of databases revealed a high prevalence of mutations in *TP53*, with splice region variants, missense variants, and intron variants being the most common. *VEGFA* and *CTH* also displayed mutations, primarily missense and intron variants. Gene expression analysis showed significantly higher levels of *TP53* and *VEGFA* in GBM patients compared to controls. *CTH* expression also exhibited a slight increase in GBM patients. Sanger sequencing identified three mutations in the *TP53* gene, including a novel mutation (11915C>A) not previously reported in external databases. Additionally, novel mutations were found in the *VEGFA* (841G>GA, 919T>TG) and *CTH* (28398A>AC, 28399A>AT) genes.

Conclusions

This study highlights the immune dysregulation, inflammation, and genetic variations in GBM. The findings emphasize the potential importance of the *TP53*, *VEGFA,* and *CTH* genes as targets for therapies and diagnostic biomarkers of GBM. Further study is necessary to comprehend these genetic variations' functional implications and their use in personalized GBM treatment.

## Introduction

Glioblastoma (GBM) is the most common and deadly primary CNS malignancy. Brain tumors and nervous system malignancies will account for 300,000 new cases and 250,000 deaths a year, 48% of CNS tumors are GBM [1]. In 2021, the WHO classified brain tumors according to genetic changes and histologic characteristics. The four types of GBM are adult-type diffuse gliomas, pediatric-type diffuse low-grade gliomas, pediatric-type diffuse high-grade gliomas, and confined astrocytic gliomas [2].

Overexpression of vascular endothelial growth factor (VEGF) is a feature of GBM. VEGF stimulates extensive angiogenesis and has a direct correlation with the malignancy and prognosis of GBM [3]. Accordingly, single nucleotide polymorphisms of this gene could play an important role in structural and functional alterations, leading to overexpression of this gene in GBM [4]. The mutant variant of *TP53* promotes tumor cell proliferation and accelerates the malignant transformation of astrocytic tumors [5]. This variation is often connected with the *IDH-1* mutation (65%-90% of cases), but the *TP53* gene mutation occurs in only 30% of *IDH *wild-type GBM [6].

Hydrogen sulfide (H_2_S) is widely recognized as a carcinogenic gas and has the potential to be a target for cancer therapy and detection due to its role in cancer progression. Some enzymes, like cystathionine β-synthase (CBS) and *CTH*, need pyridoxal 5' phosphate to do their job. These enzymes are responsible for the conversion of L-cysteine into endogenous H_2_S inside human tissues. The liver, kidney, and brain contain abundant amounts of *CTH* [7]. Different types of cancer, including prostate cancer, gastric cancer, and melanoma cells, exhibit an increase in *CTH* [8]. Our research group has found a number of clinically important mutations in colorectal cancer [9] and lung adenocarcinoma [10]. However, little research currently investigates a putative relationship between GBM-causing genes and H_2_S-producing genes. This study examines the roles and implications of numerous mutations in the *TP53*, *VEGFA*, and *CTH *genes and their relationship to GBM.

## Materials and methods

Tissue and blood collection

Blood and formalin-fixed paraffin-embedded (FFPE) tissue blocks of 18 GBM patients were collected from the Central Public Health Laboratory and Private Laboratories in Duhok and Erbil governorates, respectively. FFPE tissue samples were obtained and kept at room temperature (20-22°C) before DNA extraction and genotyping. The tumor samples from the paraffin blocks were macro-dissected. All specimens were placed in Eppendorf containers to avoid tissue cross-contamination. Also, blood samples were collected from GBM and healthy individuals for hematological and biochemical analysis.

Hematological and biochemical measurements

The total white blood cell counts and differential leukocyte counts were measured using a CBC Coulter Counter Convergys X3 (Convergent Technologies GmbH & Co. KG, Germany) as per the manufacturer's instructions. To check the levels of aspartate transaminase (AST), alanine transaminase (ALT), alkaline phosphatase (ALP), and C-reactive protein (CRP) in the blood, we used a Cobas Integra 400 Plus analyzer and an Elecsys CEA ready-to-use reagent kit from Roche Diagnostics, Basel, Switzerland. This detection technique utilizes electrochemiluminescence to quantify the immunoreactivity.

Mutational landscape and expression profiles data retrieval

The present study examined the significance of mutations occurring in the *TP53*, *VEGFA*, and *CTH* genes as potential molecular markers in GBM. We retrieved data on mutations from the International Cancer Genome Consortium (ICGC) Data Portal (https://dcc.icgc.org/) and the COSMIC Mutational Signatures (https://www.sanger.ac.uk/tool/cosmic/) to achieve this objective. The COSMIC Mutational Signatures database provided an extensive collection of somatic mutation data encompassing a variety of cancer types, including GBM. This facilitated our analysis by giving us access to a wide variety of mutation profiles. The ICGC data portal provided supplementary, curated mutation data that was specific to GBM cases, thereby boosting the comprehensiveness and precision of our mutational analysis.

Subsequently, expression data was retrieved from the Gene Expression Database of Normal and Tumour Tissues (GENT2) (http://gent2.appex.kr/gent2/). This resource was used to investigate gene expression patterns in GBM samples that were associated with *TP53*, *VEGFA*, and *CTH* genes. Using GENT2, we successfully analyzed the transcriptional activity of these genes in a wide variety of GBM specimens, generating important information regarding their possible involvement in the progression and pathogenesis of GBM.

DNA extraction and quantification

Genomic DNA was extracted from FFPE tissues of GBM using a commercially available kit, the FavorPrepTM Tissue Genomic DNA Extraction Kit (Favorgen, Taiwan), following the manufacturer’s protocols. DNA was extracted and purified from 18 specimens (FFPE of GBM tissue samples). Each sample block, 10 µm in diameter, was cut and divided into ten equal sections using a semi-automated microtome (HM 340E; Thermo Fisher Scientific, Waltham, USA). Agarose gel electrophoresis and Red-Safe staining were employed to examine the quality of the extracted DNA. The DNA content was then measured using a Thermo Scientific Nanodrop 1000 spectrophotometer (Thermo Fisher Scientific, Waltham, USA), and the purity of the DNA was determined using an optimal A260/A280 absorbance ratio of 1.8.

Genotype determination

In this study, we selected three commonly studied variations in the *TP53*, *VEGFA*, and *CTH* genes. The purified DNA was amplified individually for each genetic variant using conventional PCR on a Veriti 96-thermal cycler (Thermo Fisher Scientific, Waltham, USA) using the following primers: *TP53* forward, 5′-TCCCCCTTGCCGTCCCA-3′ and reverse, 5′-CTGGTGCAGGGGCCACGC-3′; *VEGFA* forward, 5′-CTCGGTGCTGGAATT TGATATTC-3′ and reverse, 5′-CAAAAGCAGGTCACTCACTTTGC-3′; *CTH* forward, 5′-GGACTTCTTGAGGAGTTGAAGC-3′ and reverse, 5′-ATTCTCACCTCCTTCAGAGGC-3′.

Initially, a ready-to-use master mix (ADDBIO INC., Daejeon, Korea) was used, containing Taq DNA polymerase, dNTPs, KCl, and reaction buffer. The following thermocycling conditions have been used for PCR: initial denaturation at 95°C for 5 min, followed by 35 cycles of 95°C for 30 sec; different annealing temperatures were used for each polymorphism (*TP53* at 60°C, *VEGFA* at 55°C, *CTH *at 54°C) for 30 sec; and elongation at 72°C for 1 min, followed by a final extension step at 72°C for 5 min. The PCR products for *TP53* and *CTH* were separated by 2% agarose gel electrophoresis. *VEGFA* was separated on 3% agarose gel electrophoresis, compared with the 100-bp DNA marker (GDSBio, China), and stained with a safe dye (DSView Nucleic Acid Stain, GDSBio) before being cast into the tray and visualized using a gel documentation system (UV transilluminator UVP, UK). Following the PCR procedure, the products were sent for sequencing using the same forward primers for each particular polymorphism using an automatic ABI PRISM 3130 DNA sequencer (Applied Biosystems, USA). Analysis of Sanger sequencing data was achieved using the Mutation Surveyor software (Soft Genetics, State College, USA), and the mutation result was compared with public databases, including gnomAD, dbSNP, ClinVar, and COSMIC.

Statistical analysis

For biochemical and hematological analysis, comparisons between patients with GBM and healthy individuals were performed using a Mann-Whitney test, and values were presented as medians and 25% and 75% quartiles. Whereas for gene expression retrieved from the GENT2 database, comparisons between patients with GBM and healthy individuals were performed using an unpaired t-test, and values were presented as mean±SEM. All data were subjected to normality testing, including the D'Agostino and Pearson omnibus, Shapiro-Wilk, and KS tests. The graphics, computations, and statistical analyses were generated using GraphPad Prism software (version 10; GraphPad Software, Inc.). A *p*-value of <0.05 was considered statistically significant.

Ethical considerations

The Scientific Research Division's Research Ethics Committee of the University of Duhok approved the study protocol under approval number (15092021-9-13), and all procedures contributing to this work met national and institutional human experimentation committees' ethical standards and the Declaration of Helsinki.

## Results

Patient characteristics

This study includes a cohort of 18 GBM patients and 28 healthy individuals. Among these patients, 11 (61.11%) were men and seven (38.88%) were women. Individuals who participated were categorized based on their age, with 12 (66.66%) being under the age of 50, three (16.66%) being between the ages of 51 and 60, and three (16.66%) being older than 61, while healthy individuals were 14 (50%) male and 14 (50%) female, with 17 (60.71) being under the age of 50, two (7.14%) being between the ages of 51 and 60, and nine (32.14%) being older than 61. All patients included in this study were diagnosed with GBM tumors, specifically grade IV.

Hematological and biochemical analysis

**Figure 1:**
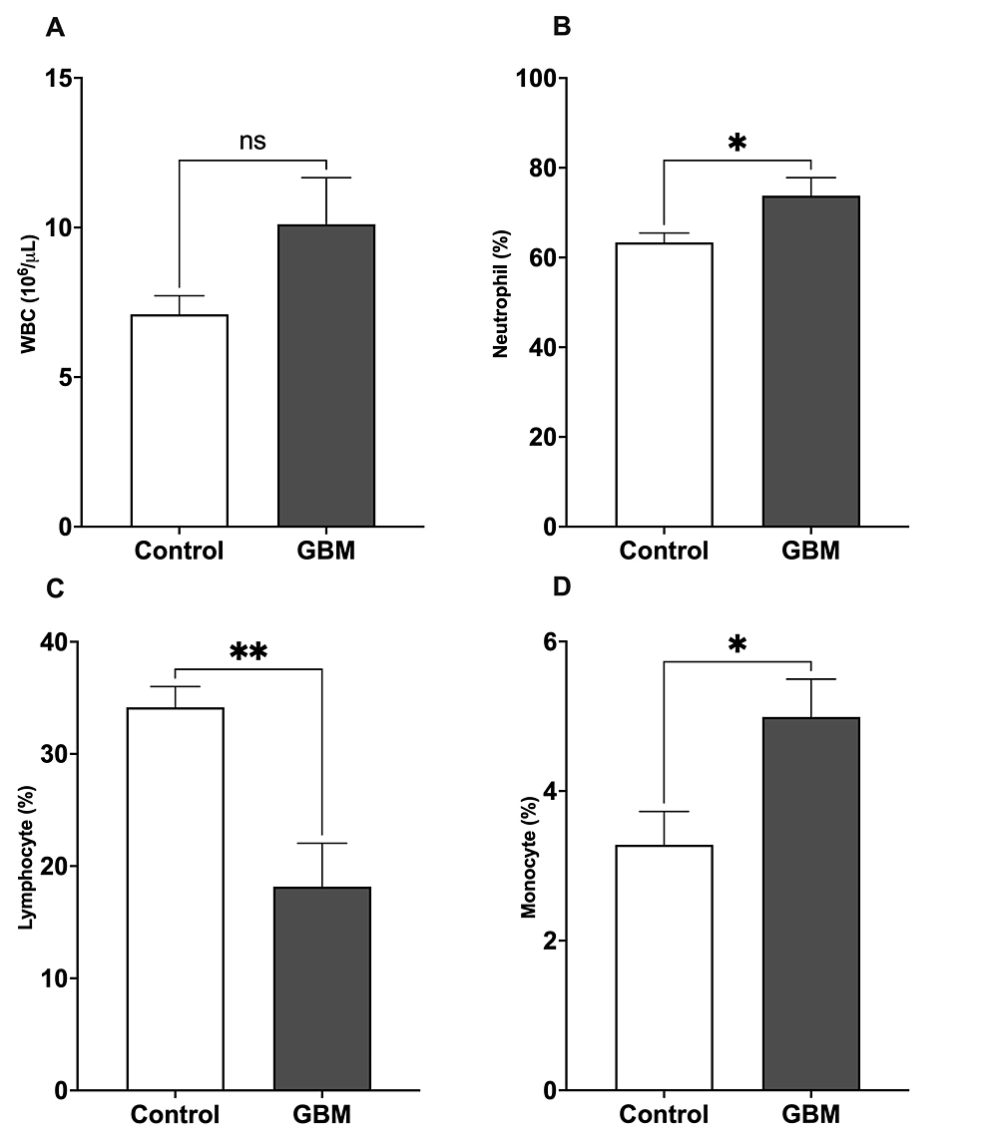
Comparison between the total number of WBCs, neutrophils, lymphocytes, and monocytes in healthy individuals and patients with GBM (A) No significant changes were observed in the total number of WBCs. (B) The percentage of neutrophils was significantly increased in patients with GBM compared to healthy individuals. (C) The percentage of lymphocytes was significantly decreased in patients with GBM compared to healthy individuals. (D) The percentage of monocytes was significantly increased in patients with GBM compared to healthy individuals. The comparison was performed using an unpaired t-test. *p<0.05; **p<0.01 vs. healthy individuals; GBM: glioblastoma; WBC: white blood cell

In the control group, the median WBC count was 6.46 (5.57, 9.13) (10^6^/µL), but in GBM patients, it was 9.1 (5.8, 14.5) (10^6^/µL). However, this difference was not statistically significant. However, WBC subgroups differed significantly. Neutrophil counts were significantly higher (*p*≤0.05) in GBM patients than in controls, with a median of 81% (63.9, 83.5) in GBM vs. 62.3% (57.2, 70) in controls. In GBM patients, lymphocyte counts were significantly lower (*p*≤0.01) than in controls, with a median of 13.4% (8.8, 28.40) in GBM vs. 34.01% (28.2, 37.8) in controls. Monocyte counts were considerably greater (*p*≤0.05) in GBM patients compared to the control median: 4.2% (3.8-7.3) in GBM vs. 3.2% (1.8-4.1) in controls (Figure 1).

**Figure 2:**
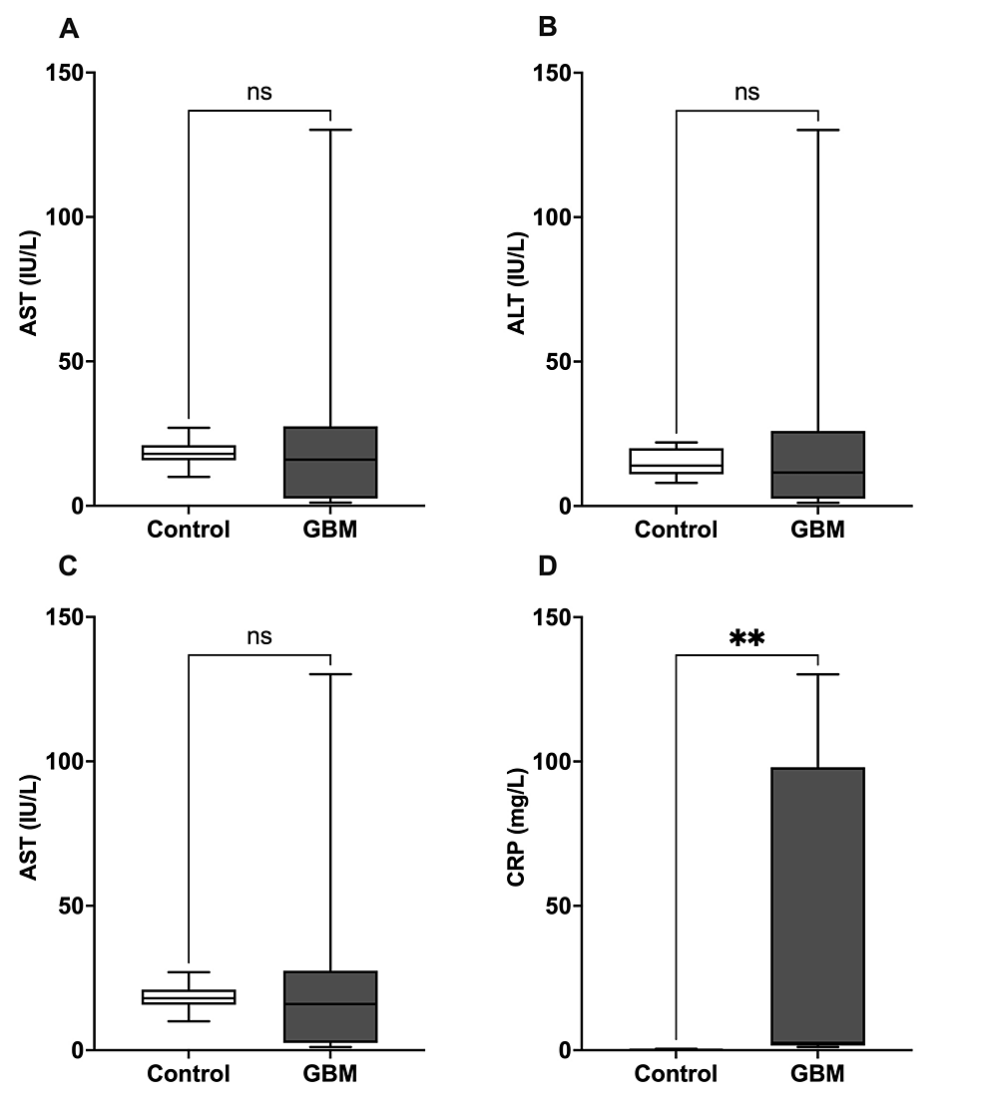
Liver function markers and CRP levels in healthy individuals and GBM patients No significant changes were observed in the AST (IU/L) (A), ALT (IU/L) (B), and ALP (IU/L) (C) in patients with GBM compared to healthy individuals. (D) The level of CRP (mg/L) was significantly increased in patients with GBM compared to healthy individuals. The comparison was performed using the Mann-Whitney test. **p<0.01 vs. healthy individuals; GBM: glioblastoma; ALT: alanine transaminase; AST: aspartate transaminase; ALP: alkaline phosphatase; CRP: C-reactive protein

The median values of AST 18 (15.75, 21) in control vs. 16 (2.55, 27.5) in GBM, ALT 14 (11, 20) in control vs. 11.6 (2.55, 26) in GBM, and ALP (61.2 (54.38, 73.03) in control vs. 68 (2.43, 138.3) in GBM) showed no statistically significant differences. GBM patients had significantly higher (P≤0.01) median CRP levels of 2.55 (1.6, 98) in GBM vs. 0.15 (0.1, 0.2) in control (Figure 2).

Mutation and expression retrieval from databases

The distribution of mutations within these genes retrieved from the gnomAD database, including various classes of mutations, is briefly presented in Table 1. A total of 2041 mutations were identified in the *TP53* gene, comprising significant mutations in the splice region variant (70), missense variant (564), and intron variant (902). *VEGFA* showed a total of 1862 mutations, with missense variant (593), intron variant (772), and synonymous variant (246) being the most prevalent. A considerable percentage of the 1839 mutations identified in *CTH* were intron variant (920), missense variant (509), and splice region variant (52).

**Table 1:** Summary of mutations in the TP53, VEGFA, and CTH genes in all types of cancer retrieved from the gnomAD database.

Genes	Total	3prime UTR variant	5prime UTR variant	Frameshift variant	Inframe deletion	Inframe insertion	Intron variant	Missense variant	Splice acceptor variant	Splice donor variant	Splice region variant	Start lost	Stop gained	Stop lost	Stop retained variant	Synonymous variant
TP53	2041	109	124	6	19	1	902	564	13	6	70	5	15	1	2	204
VEGFA	1862	64	67	39	4	2	772	593	5	8	36	3	19	3	1	246
CTH	1839	40	64	40	2	-	920	509	15	13	52	2	16	1	-	165

A summary of the mutations retrieved from the ICGC database is presented in Table 2 and Table 5 in Appendices, which classify them according to their clinical significance and impact. A total of 1079 high-impact mutations were identified in *TP53*, with 136 of them being classified as clinically significant. The majority of these mutations were composed of single-base substitutions. Among them were 25 missense mutations, one stop gained mutation, and nine likely pathogenic mutations, in addition to various pathogenic classifications: nine pathogenic, eight pathogenic/likely pathogenic, and nine likely pathogenic. Twenty-two high-impact mutations were observed in *VEGFA*, two of which were stop-gain mutations. Three of the 84 high-impact mutations identified in *CTH* were missense mutations. Notably, in this dataset, no clinically significant mutations were identified for *VEGFA* and *CTH* genes.

**Table 2:** Summary of mutations in the TP53, VEGFA, and CTH genes in GBM retrieved from the ICGC database. GBM: glioblastoma; ICGC: International Cancer Genome Consortium

Genes	High impact mutations	Clinically significant mutations	Glioblastoma	Type of mutations	Consequences	Clinical significance
TP53	1079	136	26	Single base substitution	25 Missenses, 1 Stop Gained	9 Pathogenic, 8 Pathogenic/Likely pathogenic, 9 Likely pathogenic
VEGFA	22	0	2	Single base substitution	2 Stop Gained	Non-pathogenic
CTH	84	0	3	Single base substitution	3 Missense	Non-pathogenic

The types and frequencies of COSMIC database mutations are summarized in Table 3 and Table 6 in Appendices. *TP53* had a total of 773 mutations, with the majority being substitution missense mutations (515), deletion frameshift mutations (67), and substitution nonsense mutations (46). *VEGFA* showed a lower number of mutations, including 12 mutations, consisting of five substitution missense mutations and three substitution silent mutations. *CTH *had the lowest mutation number with a total of four mutations, mostly comprising two substitution missense mutations and two unknown mutations. Notably, the data we collected did not contain complex frameshift or whole gene deletion mutations in *VEGFA* or *CTH*.

**Table 3:** Summary of the mutations in the TP53, VEGFA, and CTH genes in GBM retrieved from the COSMIC database. GBM: glioblastoma

Genes	Total	Complex frameshift	Deletion frameshift	Deletion In frame	Frameshift	Insertion frameshift	Insertion in frame	Substitution coding silent	Substitution missense	Substitution nonsense	Unknown	Whole gene deletion
TP53	773	2	67	25	21	21	6	20	515	46	49	1
VEGFA	12	-	-	-	-	-	-	2	3	2	5	-
CTH	4	-	-	-	-	-	-	-	2	-	2	-

**Figure 3:**
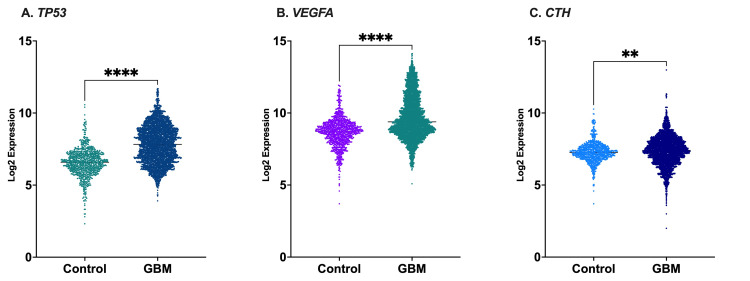
TP53, VEGFA, and CTH gene expression in healthy individuals and patients with GBM retrieved from the GENT2 database. The log2 expression values of *TP53* (A), *VEGFA* (B), and *CTH* (C) genes were significantly increased in patients with GBM compared to healthy individuals. The comparison was performed using an unpaired t-test. **p<0.01; ****p<0.001 vs. healthy individuals; GBM: glioblastoma

The analysis of gene expression in the GENT2 database showed considerable differences between the control group and patients diagnosed with GBM (Figure 3). *TP53* expression was significantly higher (*p*≤0.001) in GBM patients (7.837±0.022) than in controls (6.549±0.033). Similarly, *VEGFA* expression was significantly greater (*p*≤0.001) in GBM (9.698±0.023) than in controls (8.655±0.031). Additionally, there was a modest elevation (*p*≤0.01) in *CTH* expression in GBM patients (7.376±0.017) in comparison to the control group (7.279±0.024).

Mutation analysis

In the present study, we elected to genotype three different single nucleotide polymorphisms in the *TP53*, *VEGFA*, and *CTH *genes. Following DNA sequencing, we used a Mutation Surveyor to identify mutations in the *TP53*, *VEGFA*, and *CTH* genes (Table 4). The most nucleotide substitution that occurred in the *TP53* gene was nine times C→G. The variant mutation (11897C>G) on chromosome position (17:7579472) has been previously described in external public databases, and its variant percentage in the present study was 100%. However, the C→A substitution in the mutation variants (11915C>A) on chromosome position (17:7579454) has not been previously described in the external databases, also its variant percentage was 100%. While, the C→CT substitution in the mutation variants (11846C>CT) also occurred on chromosome position (17:7579548), causing a change in amino acid Serine to Isoleucine, in which this mutation was not found in the external databases, and its variant percentage was 12.5%.

**Table 4:** Variants identified in TP53, CTH, and VEGFA genes GBM patients analyzed with mutation DNA variant analysis. GBM: glioblastoma

Genes	Chromosome position	Exon	Mutation genotype	Mutation	Heterozygous/homozygous	Variants	Variant percentage	Amino acid change	External database
VEGF	6:43738350	1	C>CG	substitution	Heterozygous	905C>CG	37.5%	None	dbSNP:2010963
	6:43738350	1	C>G	substitution	Homozygous	905C>G	50%	None	dbSNP:2010963
	6:43738364	1	T>TG	substitution	Heterozygous	919T>TG	12.5%	None	Not found
	6:43738173	1	G>GA	substitution	Heterozygous	841G>GA	12.5%	None	Not found
TP53	17:7579472	4	C>G	substitution	Homozygous	11897C>G	100%	None	dbSNP:1042522
	17:7579454	4	C>A	substitution	Homozygous	11915C>A	100%	None	Not found
	17:7579548	4	C>CT	substitution	Heterozygous	11846C>CT	12.5%	Serine/Isoleucine	Not found
CTH	1:70904800	12	G>GT	substitution	Heterozygous	28400G>GT	25%	Serine/Isoleucine	dbSNP:1021737
	1:70904895	12	A>AG	substitution	Heterozygous	28495A>AG	25%	None	Not found
	1:70904977	12	T>TA	substitution	Heterozygous	28577T>TA	25%	None	Not found
	1:70905011	12	C>CA	substitution	Heterozygous	28611C>CA	25%	None	Not found
	1:70904798	12	A>AC	substitution	Heterozygous	28398A>AC	25%	None	Not found
	1:70904799	12	A>AT	substitution	Heterozygous	28399A>AT	25%	Serine/Cysteine	Not found
	1:70904776	12	T>TC	substitution	Heterozygous	28376T>TC	33.3%	None	Not found

In the *VEGFA* gene, heterozygous substitution and one homozygous substitution in the mutation variants (905C>CG and 905C>G) on chromosome position (6:43738350) has occurred in GBM patients, and this mutation has been previously described; its variant percentage was 37.5%; while, the G→GA substitution with mutation variant (841G>GA) that occurred on chromosome position (6:43738173), in which this mutation was not found previously in the databases, and its variant percentage was 12.5%. However, the T→TG substitution in the mutation variants (919T>TG) on chromosome position (6:43738364), in which this mutation was not found previously in the databases, and its variant percentage was 12.5%.

A total of seven mutations were identified on chromosome 1 at various positions of the *CTH* gene. Three of the newly found mutant variants in the *CTH* gene are associated with a change in amino acid level. The T→TC heterozygous substitution with mutation variant (28376T>TC) on chromosome position (1:70904776). However, four mutation variants (28400G>GT, 28495A>A, 28577T>T, and 28611C>C) on chromosome position (1:70904800, 1:70904895, 1:70904977 and 1:70905011) no amino acid changes were detected in any of them except the variants (28400G>GT,403S>S). Two mutation variants (28398A>AC and 28399A>AT) on chromosome position (1:70904798 and 1:70904799) caused a change in amino acid, nor were any of them reported in external databases, and its variant percentage for all mutations was 25%.

## Discussion

This study examined a group of 18 patients diagnosed with GBM, an extremely severe form of brain cancer. The patient group included more men than women, which is consistent with the reported higher incidence of GBM in men [11]. The majority of individuals in both the patient (66.66%) and control (60.71%) groups were under 50 years old, with fewer falling into the 51-60 and above 61 age categories. This might be attributed to the rarity of GBM in younger individuals, along with the increased death rate related to the disease in elderly people [12].

Comparing the blood parameters of GBM patients to those of a control group shows that the development of GBM is linked to significant changes in immune cell profiles and signs of inflammation. GBM patients significantly increased their neutrophil levels, indicating the presence of a pro-inflammatory condition in the tumor microenvironment [13]. In contrast, GBM patients had substantially lower lymphocyte numbers, indicating the presence of immune suppression or evasion mechanisms used by the tumor [14]. Furthermore, the number of monocytes was considerably greater in individuals with GBM, which may indicate the presence of tumor-associated macrophages infiltrating the tumor [15]. In addition, the GBM immunological microenvironment is inhabited by myeloid cells, including tumor-associated macrophages, myeloid-derived suppressor cells, neutrophils, and dendritic cells, indicating an increase in myeloid progenitors in GBM [16]. Moreover, the markedly elevated levels of CRP in individuals with GBM indicate the presence of systemic inflammation associated with the tumor [17]. Although there were changes in the immune system, the liver function markers AST, ALT, and ALP did not show any statistically significant changes between GBM patients and controls. Sarganas et al. [18] showed a substantial increase in liver function markers after temozolomide therapy. These results emphasize the complicated connection between immune dysregulation and inflammatory processes in GBM and highlight the potential diagnostic and therapeutic significance of monitoring immunological and inflammatory markers in GBM patients.

An in-depth examination of mutations retrieved from multiple databases provides essential information on the molecular basis of cancer, with a specific emphasis on essential genes, including *TP53*, *VEGFA*, and *CTH* genes. The gnomAD database revealed significant variations in the distribution of mutations across these genes, emphasizing the broad spectrum of mutation types and their incidences. An extensive number of mutations, such as splice region variants, missense variants, and intron variants, were found in the *TP53* gene, indicating the complicated sequence of changes in this gene responsible for suppressing tumor growth. Variations in missense and intron mutations were frequently observed in the *VEGFA* and *CTH* genes, showcasing a diverse array of mutation patterns. The ICGC database uncovered several significant mutations in the *TP53* gene, highlighting its importance in cancer development. In contrast, the *VEGFA* and *CTH* genes exhibited fewer variations, but there were noticeable differences in the kinds of mutations. Notably, there were no significant mutations found for the *VEGFA* and *CTH* genes in this dataset that had clinical significance. *TP53* mutations were very widespread in the COSMIC database, exhibiting a range of mutation types such as substitution missense and deletion frameshift mutations. This indicates that the gene *TP53* displays genetic heterogeneity in cancer. *VEGFA* and *CTH *genes had a lower number of alterations in comparison to *TP53*, primarily consisting of missense mutations. The current set of *VEGFA* and *CTH* genes did not contain any complex frameshift or whole gene deletion mutations. In general, these results provide significant knowledge about the genetic mutations in cancer-related genes and could direct future studies on targeted therapies and diagnostic approaches.

The analysis of genetic alterations from databases provided a roadmap for targeted sequencing in the second part of the study. The databases indicated the *TP53* gene as a critical gene with frequent mutations, which led to further analysis in GBM patients. In these individuals, *TP53* mutations, including potentially new ones, were actually found. Similarly, databases have shown possible roles for the *VEGFA* and *CTH* genes, but their changes seem to be less significant. Therefore, the current study examined the occurrence of mutations in *TP53*, *VEGFA*, and *CTH*, genes that are known to be associated with the development of GBM. The study identified numerous mutations in all three genes, providing valuable information on the possible genetic characteristics of GBM in the population under consideration.

The *TP53* gene, which acts as a tumor suppressor, plays a crucial role in preserving the integrity of the genome and inhibiting excessive cell proliferation [19]. Several types of malignancies, including GBM, frequently exhibit *TP53* mutations [20]. The investigation of the *TP53* gene in GBM patients provided curious findings regarding specific mutations and their frequency. In total, three mutations in the *TP53* gene of GBM patients were identified in three variants at various positions on chromosome 17. A noteworthy finding was the high frequency of C→G nucleotide substitutions in the *TP53* gene, namely, the variation 11897C>G (chromosome 17:7579472), which was present in all GBM cases. This specific mutation has been recorded in external databases, confirming prior findings of its connection with the development of GBM [21]. The 11897C>G mutation in our study cohort highlights its potential importance in GBM development. In addition, we found two previously unidentified mutations in the TP53 gene: 11915C>A, which is present in 100% of cases, and 11846C>CT, which has a frequency of 12.5%. External databases have not reported the 11915C>A mutation, suggesting the possibility of discovering a new variation. Similarly, the mutation 11846C>CT, while less common, results in an alteration of the amino acid sequence from Serine to isoleucine. The functional implications of these new mutations are still unclear. More studies are needed to determine their impact on the structure and function of the p53 protein, as well as their possible role in the development of GBM.

VEGF plays a crucial role in angiogenesis, and the growth and progression of various malignancies, including GBM, are associated with elevated *VEGFA* expression [4]. The most frequently detected mutation was a hybrid of heterozygous and homozygous substitutions at locations 905C>CG and 905C>G on chromosome 6:43738350. We observed this mutation in colorectal cancer in our previous research but not in GMB [22]. Mutations in this specific location of the *VEGFA* gene have the potential to affect the signaling pathways and stimulate the growth of blood vessels in the GBM. The observed prevalence of 37.5% indicates that this mutation could have significant consequences for a significant number of GBM patients. We have also identified two new mutations in the *VEGFA* gene: 841G>GA (chromosome 6: 43738173) and 919T>TG (chromosome 6: 43738364). Both mutations have a prevalence rate of 12.5%. These mutations have not been previously reported in external databases. The effects of these new mutations on the structure and function of the VEGF protein are yet unclear. Therefore, these findings provide an opportunity to explore novel therapies for GBM.

The involvement of mutations in the *CTH* gene has been proposed in several types of cancer [9,10], but their precise contribution to the formation of GBM is still not well understood. The study of the *CTH* gene in GBM patients identified a combination of silent mutations and mutations that result in changes to amino acids. Amino acid substitutions occurred in three out of the seven identified mutations: 28376T>TC, 28398A>AC, and 28399A>AT. The lack of prior documentation in external databases regarding these mutations underscores the possibility of finding new variants; the functional implications of these amino acid substitutions remain unknown. The remaining four mutations (28495A>A, 284400G>GT, 28577T>T, and 28611C>C) were silent mutations. Despite their generally lower probability of significantly affecting protein function, silent mutations can still influence splicing, mRNA stability, or protein expression levels [23].

The GENT2 database's analysis of gene expression showed a clear and distinct trend in GBM patients, particularly the elevated expression of *TP53* and *VEGFA *genes. This finding seems paradoxical for *TP53*, a gene that typically inhibits tumor growth and is usually suppressed in cancer [24]. However, cellular stress or DNA damage can upregulate *TP53* despite the presence of inactivating mutations that render it non-functional [25,26]. To understand the role of *TP53* in the current scenario, additional studies into its mutational state and how it works within GBM patients are required. However, there is widespread recognition that GBM elevates *VEGFA* [27]. This finding is consistent with previous investigations and emphasizes the continual importance of *VEGFA* signaling pathways as possible targets for treatment in GBM [28]. *CTH* expression in GBM showed a modest but statistically significant increase. Although the exact function of *CTH* in GBM is still unclear, its possible significance in cell adhesion and migration justifies additional investigation, especially in connection with GBM invasion [29]. These findings indicate an intricate interaction between variations in gene expression and the progression of GBM; therefore, further study is required to examine the practical effects of these alterations in expression and their potential as targets for medical treatment.

The study's limitations include a restricted GBM patient sample, which may limit the application of the findings to larger groups. Furthermore, the study failed to account for possible confounding factors such as comorbidities or medication usage, which might explain the observed variations in blood parameters and genetic abnormalities.

## Conclusions

This study identified substantial changes in immune cell composition and inflammatory marker levels in GBM patients compared to the control group. The findings suggest a tumor microenvironment in GBM that promotes inflammation and suppresses immunological responses. Gene expression study found higher *TP53 *and *VEGFA *activity in GBM patients, possibly attributable to non-functional mutations. Further examination into the *TP53 *mutational status is required. The work underscores the relevance of *VEGFA *signaling pathways as therapeutic targets in GBM. *CTH *expression showed a modest increase in GBM, hinting at its potential role in GBM formation, necessitating further research. In conclusion, this study provides insight into the complex relationships between immune dysregulation, inflammation, and gene expression changes in GBM, opening the way for future strategies for therapy.

## Appendices

**Table 5:** Mutations in the TP53, VEGFA, and CTH genes in GBM retrieved from the ICGC database. GBM: glioblastoma; ICGC: International Cancer Genome Consortium

TP53	Mutation ID	Genomic DNA change	Type	Consequences
1	MU7870	chr17:g.7578406C>T	single base substitution	Missense: TP53 R82H, R175H, R43H|Upstream: TP53 |Exon: TP53 |Downstream: TP53
2	MU17943	chr17:g.7577539G>A	single base substitution	Missense: TP53 R248W, R116W, R155W|Upstream: TP53 |Exon: TP53 |Downstream: TP53
3	MU4807	chr17:g.7578190T>C	single base substitution	Missense: TP53 Y127C, Y88C, Y220C|Upstream: TP53 |Exon: TP53 |Downstream: TP53 |Intron: TP53
4	MU25838	chr17:g.7577548C>T	single base substitution	Missense: TP53 G113S, G152S, G245S|Upstream: TP53 |Exon: TP53 |Downstream: TP53
5	MU26668	chr17:g.7577568C>A	single base substitution	Missense: TP53 C238F, C145F, C106F|Upstream: TP53 |Exon: TP53 |Downstream: TP53
6	MU8496	chr17:g.7578265A>G	single base substitution	Missense: TP53 I195T, I63T, I102T|Upstream: TP53 |Exon: TP53 |Downstream: TP53 |Intron: TP53
7	MU599284	chr17:g.7578211C>T	single base substitution	Missense: TP53 R213Q, R81Q, R120Q|Upstream: TP53 |Exon: TP53 |Downstream: TP53 |Intron: TP53
8	MU17011	chr17:g.7577022G>A	single base substitution	Stop Gained: TP53 R306*, R174*|Upstream: TP53 |Exon: TP53 |Downstream: TP53 |Intron: TP53
9	MU607204	chr17:g.7577556C>T	single base substitution	Missense: TP53 C110Y, C242Y, C149Y|Upstream: TP53 |Exon: TP53 |Downstream: TP53
10	MU30748	chr17:g.7577538C>T	single base substitution	Missense: TP53 R116Q, R155Q, R248Q|Upstream: TP53 |Exon: TP53 |Downstream: TP53
11	MU24637	chr17:g.7577120C>T	single base substitution	Missense: TP53 R141H, R273H|Upstream: TP53 |Exon: TP53 |Downstream: TP53 |Intron: TP53
12	MU117000	chr17:g.7577114C>T	single base substitution	Missense: TP53 C143Y, C275Y|Upstream: TP53 |Exon: TP53 |Downstream: TP53 |Intron: TP53
13	MU11094	chr17:g.7577568C>T	single base substitution	Missense: TP53 C238Y, C145Y, C106Y|Upstream: TP53 |Exon: TP53 |Downstream: TP53
14	MU7717	chr17:g.7577580T>C	single base substitution	Missense: TP53 Y234C, Y141C, Y102C|Upstream: TP53 |Exon: TP53 |Downstream: TP53
15	MU831551	chr17:g.7577550C>T	single base substitution	Missense: TP53 G112D, G151D, G244D|Upstream: TP53 |Exon: TP53 |Downstream: TP53
16	MU35740	chr17:g.7578395G>A	single base substitution	Missense: TP53 H47Y, H179Y, H86Y|Upstream: TP53 |Exon: TP53 |Downstream: TP53
17	MU605998	chr17:g.7577551C>T	single base substitution	Missense: TP53 G244S, G151S, G112S|Upstream: TP53 |Exon: TP53 |Downstream: TP53
18	MU588945	chr17:g.7577096T>G	single base substitution	Missense: TP53 D281A, D149A|Upstream: TP53 |Exon: TP53 |Downstream: TP53 |Intron: TP53
19	MU588536	chr17:g.7577097C>G	single base substitution	Missense: TP53 D281H, D149H|Upstream: TP53 |Exon: TP53 |Downstream: TP53 |Intron: TP53
20	MU1664156	chr17:g.7577559G>T	single base substitution	Missense: TP53 S241Y, S148Y, S109Y|Upstream: TP53 |Exon: TP53 |Downstream: TP53
21	MU604332	chr17:g.7577538C>A	single base substitution	Missense: TP53 R248L, R116L, R155L|Upstream: TP53 |Exon: TP53 |Downstream: TP53
22	MU13250	chr17:g.7578271T>C	single base substitution	Missense: TP53 H193R, H61R, H100R|Upstream: TP53 |Exon: TP53 |Downstream: TP53 |Intron: TP53
23	MU1384978	chr17:g.7577560A>G	single base substitution	Missense: TP53 S241P, S148P, S109P|Upstream: TP53 |Exon: TP53 |Downstream: TP53
24	MU1382	chr17:g.7577517A>C	single base substitution	Missense: TP53 I123S, I255S|Upstream: TP53 |Exon: TP53 |Downstream: TP53
25	MU603634	chr17:g.7577535C>G	single base substitution	Missense: TP53 R117T, R249T|Upstream: TP53 |Synonymous: TP53 ?156|Exon: TP53 |Downstream: TP53
26	MU17274	chr17:g.7578235T>C	single base substitution	Missense: TP53 Y73C, Y205C, Y112C|Upstream: TP53 |Exon: TP53 |Downstream: TP53 |Intron: TP53
VEGFA	Mutation ID	Genomic DNA Change	Type	Consequences
1	MU129112547	chr6:g.43749699T>A	single base substitution	Stop Gained: VEGFA C143*, C341*, C184*, C364*, C115*, C347*, C161*, C167*, C323*|Exon: VEGFA |Downstream: VEGFA |Intron: VEGFA
2	MU591429	chr6:g.43748479C>T	single base substitution	Stop Gained: VEGFA R325*, R145*, R296*|Exon: VEGFA |Downstream: VEGFA |Intron: VEGFA
CTH	Mutation ID	Genomic DNA Change	Type	Consequences
1	MU129034369	chr1:g.70900841G>A	single base substitution	Missense: CTH E313K, E345K, E301K|Exon: CTH |Downstream: CTH
2	MU129069008	chr1:g.70904409G>T	single base substitution	Missense: CTH R332I, R320I, R364I|Exon: CTH
3	MU130816925	chr1:g.70883695G>A	single base substitution	Missense: CTH C109Y|Intron: CTH

**Table 6:** Mutations in the TP53, VEGFA, and CTH genes in GBM retrieved from the COSMIC database. GBM: glioblastoma

TP53	Position	CDS mutation	AA mutation	Legacy mutation ID	Count	Type
1	19	c.55_59delinsA	p.F19Kfs*24	COSM6943854	1	Complex - frameshift
2	339	c.1016delinsGG	p.E339Gfs*8	COSM10073065	1	Complex - frameshift
3	32	c.93del	p.L32Cfs*12	COSM5048959	1	Deletion - Frameshift
4	47	c.140_162del	p.P47Hfs*2	COSM4968945	1	Deletion - Frameshift
5	69	c.205del	p.A69Lfs*54	COSM39408	1	Deletion - Frameshift
6	80	c.239del	p.P80Lfs*43	COSM45219	1	Deletion - Frameshift
7	89	c.266_267del	p.P89Lfs*59	COSM10073066	1	Deletion - Frameshift
8	90	c.267del	p.S90Pfs*33	COSM18610	2	Deletion - Frameshift
9	112	c.335del	p.G112Afs*11	COSM6196473	1	Deletion - Frameshift
10	122	c.365_366del	p.V122Dfs*26	COSM44882	1	Deletion - Frameshift
11	131	c.390_426del	p.N131Cfs*27	COSM44576	2	Deletion - Frameshift
12	137	c.409del	p.L137Wfs*33	COSM45058	1	Deletion - Frameshift
13	139	c.417_418del	p.K139Nfs*9	COSM35650	1	Deletion - Frameshift
14	150	c.447_459del	p.T150Afs*16	COSM5752221	1	Deletion - Frameshift
15	152	c.455_459del	p.P152Rfs*27	COSM507584	1	Deletion - Frameshift
16	155	c.462_474del	p.T155Pfs*11	COSM10077562	1	Deletion - Frameshift
17	156	c.466del	p.R156Afs*14	COSM43691	1	Deletion - Frameshift
18	158	c.472del	p.R158Afs*12	COSM43781	1	Deletion - Frameshift
19	158	c.473_476del	p.R158Pfs*11	COSM43831	2	Deletion - Frameshift
20	160	c.478del	p.M160Wfs*10	COSM45112	1	Deletion - Frameshift
21	161	c.481del	p.A161Pfs*9	COSM44230	1	Deletion - Frameshift
22	164	c.491_494del	p.K164Sfs*5	COSM44339	1	Deletion - Frameshift
23	164	c.491del	p.K164Sfs*6	COSM44861	1	Deletion - Frameshift
24	167	c.499del	p.Q167Sfs*3	COSM44336	1	Deletion - Frameshift
25	170	c.508_514del	p.T170Lfs*2	COSM39398	1	Deletion - Frameshift
26	178	c.532del	p.H178Tfs*69	COSM43978	1	Deletion - Frameshift
27	183	c.547_548del	p.S183Rfs*2	COSM3734699	1	Deletion - Frameshift
28	185	c.553del	p.S185Afs*62	COSM45314	1	Deletion - Frameshift
29	188	c.563del	p.L188Rfs*59	COSM6979975	1	Deletion - Frameshift
30	190	c.567_586del	p.P190Sfs*12	COSM6966898	1	Deletion - Frameshift
31	190	c.569del	p.P190Lfs*57	COSM44454	1	Deletion - Frameshift
32	195	c.584del	p.I195Tfs*52	COSM6201897	2	Deletion - Frameshift
33	196	c.586del	p.R196Efs*51	COSM44757	1	Deletion - Frameshift
34	199	c.595_599del	p.G199Ffs*8	COSM4970228	1	Deletion - Frameshift
35	200	c.599del	p.N200Ifs*47	COSM43859	1	Deletion - Frameshift
36	205	c.613del	p.Y205Ifs*42	COSM45525	1	Deletion - Frameshift
37	206	c.617del	p.L206Wfs*41	COSM44852	1	Deletion - Frameshift
38	209	c.626_627del	p.R209Kfs*6	COSM6482	5	Deletion - Frameshift
39	211	c.631del	p.T211Lfs*36	COSM44371	1	Deletion - Frameshift
40	212	c.635_636del	p.F212Sfs*3	COSM44162	2	Deletion - Frameshift
41	213	c.636del	p.R213Dfs*34	COSM44358	2	Deletion - Frameshift
42	213	c.637del	p.R213Dfs*34	COSM43807	1	Deletion - Frameshift
43	214	c.641del	p.H214Lfs*33	COSM128666	1	Deletion - Frameshift
44	214	c.642_643del	p.H214Qfs*7	COSM46269	2	Deletion - Frameshift
45	220	c.657del	p.Y220Mfs*27	COSM44585	1	Deletion - Frameshift
46	222	c.665del	p.P222Rfs*25	COSM44270	1	Deletion - Frameshift
47	231	c.691del	p.T231Pfs*16	COSM45784	1	Deletion - Frameshift
48	234	c.699_705del	p.Y234Tfs*11	COSM1626221	2	Deletion - Frameshift
49	235	c.703_704del	p.N235Lfs*4	COSM4966998	1	Deletion - Frameshift
50	239	c.716del	p.N239Tfs*8	COSM44183	1	Deletion - Frameshift
51	245	c.734del	p.G245Afs*2	COSM44642	1	Deletion - Frameshift
52	256	c.766del	p.T256Hfs*89	COSM45526	3	Deletion - Frameshift
53	256	c.767del	p.T256Nfs*89	COSM508592	1	Deletion - Frameshift
54	262	c.785del	p.G262Vfs*83	COSM44697	2	Deletion - Frameshift
55	267	c.798del	p.R267Gfs*78	COSM6912585	1	Deletion - Frameshift
56	276	c.827_830del	p.A276Vfs*68	COSM44342	1	Deletion - Frameshift
57	285	c.854_855del	p.E285Gfs*20	COSM44337	1	Deletion - Frameshift
58	288	c.863del	p.N288Ifs*57	COSM45459	1	Deletion - Frameshift
59	294	c.880del	p.E294Sfs*51	COSM6621	1	Deletion - Frameshift
60	301	c.902del	p.P301Qfs*44	COSM45184	1	Deletion - Frameshift
61	306	c.916del	p.R306Efs*39	COSM44631	1	Deletion - Frameshift
62	313	c.936del	p.S313Afs*32	COSM45392	1	Deletion - Frameshift
63	333	c.997del	p.R333Vfs*12	COSM69084	1	Deletion - Frameshift
64	336	c.1005_1006del	p.E336Afs*10	COSM1268340	2	Deletion - Frameshift
65	339	c.1017_1018del	p.E339Dfs*7	COSM9854012	1	Deletion - Frameshift
66	342	c.1025del	p.R342Qfs*3	COSM46154	1	Deletion - Frameshift
67	349	c.1045del	p.E349Nfs*21	COSM46056	2	Deletion - Frameshift
68	376	c.1125_1128del	p.S376Pfs*45	COSM6944989	1	Deletion - Frameshift
69	382	c.1146del	p.K382Nfs*40	COSM13747	2	Deletion - Frameshift
70	57	c.169_228del	p.D57_A76del	COSM46324	1	Deletion - In frame
71	101	c.303_308del	p.K101_Y103delinsN	COSM255314	1	Deletion - In frame
72	113	c.339_341del	p.F113del	COSM45143	1	Deletion - In frame
73	126	c.376_396del	p.Y126_K132del	COSM44405	1	Deletion - In frame
74	129	c.385_387del	p.A129del	COSM44947	1	Deletion - In frame
75	131	c.393_395del	p.N131del	COSM44212	5	Deletion - In frame
76	131	c.?	p.N131del	COSM5991646	1	Deletion - In frame
77	153	c.450_452del	p.P153del	COSM6920004	1	Deletion - In frame
78	155	c.463_468del	p.T155_R156del	COSM46355	4	Deletion - In frame
79	157	c.470_475del	p.V157_R158del	COSM43727	2	Deletion - In frame
80	177	c.529_546del	p.P177_C182del	COSM43570	2	Deletion - In frame
81	179	c.534_536del	p.H179del	COSM45560	1	Deletion - In frame
82	187	c.559_561delGGT	p.G187del	COSM144169	1	Deletion - In frame
83	191	c.572_574del	p.P191del	COSM44234	3	Deletion - In frame
84	195	c.584_598del	p.I195_G199del	COSM44534	1	Deletion - In frame
85	225	c.674_682del	p.V225_S227del	COSM45452	1	Deletion - In frame
86	235	c.704_709del	p.N235_Y236del	COSM44333	1	Deletion - In frame
87	236	c.706_708del	p.Y236del	COSM44072	1	Deletion - In frame
88	238	c.712_714del	p.C238del	COSM46322	1	Deletion - In frame
89	239	c.716_721del	p.N239_S241delinsT	COSM4302130	1	Deletion - In frame
90	248	c.742_750del	p.R248_P250del	COSM44533	1	Deletion - In frame
91	252	c.754_756del	p.L252del	COSM44247	1	Deletion - In frame
92	255	c.764_766del	p.I255del	COSM43694	7	Deletion - In frame
93	265	c.792_794del	p.L265del	COSM45150	4	Deletion - In frame
94	337	c.1010_1018del	p.R337_M340delinsL	COSM4745947	1	Deletion - In frame
95	47	c.?	p.P47fs*?	COSM7002268	1	Frameshift
96	52	c.?	p.Q52Xfs*71	COSM3734733	2	Frameshift
97	64	c.?	p.P64Xfs*58	COSM4745856	1	Frameshift
98	68	c.?	p.E68Xfs*54	COSM4745853	1	Frameshift
99	93	c.?	p.L93Cfs*30	COSM6024076	1	Frameshift
100	121	c.?	p.S121fs*?	COSM7002259	1	Frameshift
101	122	c.?	p.V122Xfs*25	COSM4745854	1	Frameshift
102	150	c.?	p.T150fs*?	COSM7002261	1	Frameshift
103	152	c.?	p.P152Rfs*18	COSM9114970	1	Frameshift
104	152	c.?	p.P152Xfs*18	COSM4603663	1	Frameshift
105	174	c.?	p.R174*	COSM4603631	2	Frameshift
106	199	c.?	p.G199fs*?	COSM7002260	1	Frameshift
107	209	c.?	p.R209Xfs*6	COSM5945684	2	Frameshift
108	215	c.?	p.S215Xfs*?	COSM1169531	1	Frameshift
109	218	c.?	p.V218Gfs*4	COSM7002371	1	Frameshift
110	237	c.?	p.M237Ifs*10	COSM6024042	1	Frameshift
111	260	c.?	p.S260fs*3	COSM4745861	1	Frameshift
112	278	c.?	p.P278Lfs*67	COSM6024043	1	Frameshift
113	284	c.?	p.T284fs*?	COSM5945723	1	Frameshift
114	290	c.?	p.R290Qfs*15	COSM6024041	1	Frameshift
115	305	c.?	p.K305Xfs*40	COSM4766151	1	Frameshift
116	42	c.116_123dup	p.D42Qfs*5	COSM6948685	1	Insertion - Frameshift
117	97	c.289dup	p.V97Gfs*52	COSM10077571	1	Insertion - Frameshift
118	122	c.363dup	p.V122Cfs*27	COSM8266503	2	Insertion - Frameshift
119	124	c.372del	p.C124*	COSM69020	1	Insertion - Frameshift
120	133	c.383_396dup	p.M133Lfs*42	COSM6968586	1	Insertion - Frameshift
121	139	c.414_415insNNNN	p.K139fs*11	COSM46321	1	Insertion - Frameshift
122	146	c.434_435insNN	p.W146fs*25	COSM46262	1	Insertion - Frameshift
123	171	c.510_511dup	p.E171Gfs*4	COSM7481695	1	Insertion - Frameshift
124	173	c.516_517insN	p.V173fs*8	COSM43546	1	Insertion - Frameshift
125	176	c.528_541delinsA	p.C176*	COSM6925117	1	Insertion - Frameshift
126	182	c.542_543dup	p.C182Afs*66	COSM4971129	1	Insertion - Frameshift
127	193	c.575dup	p.H193Afs*16	COSM10536703	6	Insertion - Frameshift
128	217	c.648_649insNNNNNNNN	p.V217fs*33	COSM46216	1	Insertion - Frameshift
129	218	c.652dup	p.V218Gfs*4	COSM6969098	1	Insertion - Frameshift
130	239	c.714_715insN	p.N239fs*25	COSM44155	2	Insertion - Frameshift
131	261	c.769_781dup	p.S261Tfs*7	COSM9869125	1	Insertion - Frameshift
132	302	c.902dup	p.G302Rfs*4	COSM4745930	1	Insertion - Frameshift
133	308	c.895_919dup	p.L308Afs*6	COSM6934703	1	Insertion - Frameshift
134	308	c.920-3_920dup	p.L308Sfs*30	COSM6923871	1	Insertion - Frameshift
135	316	c.945_946dup	p.P316Lfs*30	COSM6964186	1	Insertion - Frameshift
136	342	c.1024del	p.R342Efs*3	COSM18597	3	Insertion - Frameshift
137	142	c.423_425dup	p.P142dup	COSM6934393	1	Insertion - In frame
138	157	c.469_471dup	p.V157dup	COSM144168	1	Insertion - In frame
139	184	c.548_549insGCCCCCACCATGAGCGCTGCT	p.S183_D184insPPP*ALL	COSM674055	1	Insertion - In frame
140	232	c.680_694dup	p.T231_I232insTDCTT	COSM6974264	1	Insertion - In frame
141	238	c.714_715insNNN	p.C238_N239insX	COSM44532	1	Insertion - In frame
142	254	c.759_760insCTC	p.T253_I254insL	COSM10077583	1	Insertion - In frame
143	84	c.252C>T	p.A84=	COSM45512	1	Substitution - coding silent
144	125	c.375G>A	p.T125=	COSM43904	5	Substitution - coding silent
145	125	c.375G>T	p.T125=	COSM45940	1	Substitution - coding silent
146	126	c.378C>T	p.Y126=	COSM44196	1	Substitution - coding silent
147	137	c.411G>A	p.L137=	COSM44649	1	Substitution - coding silent
148	149	c.447C>T	p.S149=	COSM44408	1	Substitution - coding silent
149	157	c.471C>A	p.V157=	COSM43934	1	Substitution - coding silent
150	173	c.519G>T	p.V173=	COSM43752	1	Substitution - coding silent
151	177	c.531C>T	p.P177=	COSM43679	2	Substitution - coding silent
152	196	c.586C>A	p.R196=	COSM44615	1	Substitution - coding silent
153	213	c.637C>A	p.R213=	COSM43798	1	Substitution - coding silent
154	213	c.639A>G	p.R213=	COSM249885	2	Substitution - coding silent
155	224	c.672G>A	p.E224=	COSM44754	1	Substitution - coding silent
156	248	c.742C>A	p.R248=	COSM44920	1	Substitution - coding silent
157	261	c.783T>C	p.S261=	COSM43748	1	Substitution - coding silent
158	265	c.795G>A	p.L265=	COSM43785	1	Substitution - coding silent
159	301	c.903A>G	p.P301=	COSM44165	1	Substitution - coding silent
160	306	c.918A>T	p.R306=	COSM43745	1	Substitution - coding silent
161	320	c.960G>A	p.K320=	COSM44197	1	Substitution - coding silent
162	324	c.972T>C	p.D324=	COSM45815	1	Substitution - coding silent
163	3	c.?	p.E3G	COSM5945685	1	Substitution - Missense
164	10	c.29T>G	p.V10G	COSM510151	1	Substitution - Missense
165	27	c.?	p.P27L	COSM5945686	1	Substitution - Missense
166	49	c.145G>A	p.D49N	COSM305601	1	Substitution - Missense
167	60	c.178C>G	p.P60A	COSM39514	1	Substitution - Missense
168	60	c.179C>G	p.P60R	COSM39453	1	Substitution - Missense
169	64	c.190C>T	p.P64S	COSM6976875	1	Substitution - Missense
170	65	c.?	p.R65K	COSM4169574	1	Substitution - Missense
171	67	c.199C>T	p.P67S	COSM44199	1	Substitution - Missense
172	68	c.203A>G	p.E68G	COSM44790	1	Substitution - Missense
173	72	c.215C>G	p.P72R	COSM250061	3	Substitution - Missense
174	72	c.?	p.P72R	COSM5346903	1	Substitution - Missense
175	73	c.218T>A	p.V73E	COSM44556	1	Substitution - Missense
176	74	c.?	p.A74V	COSM221572	1	Substitution - Missense
177	76	c.?	p.A76T	COSM221566	1	Substitution - Missense
178	81	c.242C>T	p.T81I	COSM44200	1	Substitution - Missense
179	84	c.251C>T	p.A84V	COSM44194	2	Substitution - Missense
180	84	c.?	p.A84V	COSM221567	1	Substitution - Missense
181	85	c.253C>T	p.P85S	COSM45918	2	Substitution - Missense
182	85	c.?	p.P85L	COSM6849516	1	Substitution - Missense
183	86	c.?	p.A86E	COSM4169575	1	Substitution - Missense
184	86	c.?	p.A86S	COSM221569	1	Substitution - Missense
185	87	c.260C>A	p.P87Q	COSM43544	1	Substitution - Missense
186	98	c.293C>T	p.P98L	COSM44681	1	Substitution - Missense
187	98	c.?	p.P98L	COSM4169573	1	Substitution - Missense
188	105	c.313G>A	p.G105S	COSM78687	1	Substitution - Missense
189	105	c.313G>C	p.G105R	COSM45179	3	Substitution - Missense
190	105	c.?	p.G105S	COSM1666889	1	Substitution - Missense
191	106	c.?	p.S106R	COSM4745858	1	Substitution - Missense
192	109	c.325T>G	p.F109V	COSM48817	2	Substitution - Missense
193	109	c.326T>C	p.F109S	COSM45169	1	Substitution - Missense
194	110	c.328C>T	p.R110C	COSM43682	1	Substitution - Missense
195	110	c.329G>C	p.R110P	COSM11250	2	Substitution - Missense
196	110	c.329G>T	p.R110L	COSM10716	2	Substitution - Missense
197	111	c.331C>A	p.L111M	COSM43790	1	Substitution - Missense
198	111	c.332T>A	p.L111Q	COSM44630	1	Substitution - Missense
199	111	c.332T>C	p.L111P	COSM44045	1	Substitution - Missense
200	111	c.332T>G	p.L111R	COSM44570	1	Substitution - Missense
201	111	c.?	p.L111P	COSM5033525	1	Substitution - Missense
202	113	c.337T>G	p.F113V	COSM11498	2	Substitution - Missense
203	113	c.?	p.F113L	COSM1169535	2	Substitution - Missense
204	120	c.358A>G	p.K120E	COSM44827	3	Substitution - Missense
205	120	c.359A>T	p.K120M	COSM44190	2	Substitution - Missense
206	120	c.?	p.K120E	COSM4745863	2	Substitution - Missense
207	123	c.368C>T	p.T123I	COSM44188	1	Substitution - Missense
208	124	c.370T>C	p.C124R	COSM11147	1	Substitution - Missense
209	124	c.?	p.C124R	COSM5033526	2	Substitution - Missense
210	125	c.373A>C	p.T125P	COSM45368	1	Substitution - Missense
211	125	c.374C>G	p.T125R	COSM45243	4	Substitution - Missense
212	125	c.374C>T	p.T125M	COSM44988	1	Substitution - Missense
213	125	c.?	p.T125R	COSM4745855	1	Substitution - Missense
214	126	c.376T>A	p.Y126N	COSM44380	2	Substitution - Missense
215	126	c.376T>G	p.Y126D	COSM43900	2	Substitution - Missense
216	126	c.377A>G	p.Y126C	COSM11517	1	Substitution - Missense
217	126	c.?	p.Y126H	COSM4169572	1	Substitution - Missense
218	127	c.379T>A	p.S127T	COSM53285	1	Substitution - Missense
219	127	c.380C>A	p.S127Y	COSM43970	4	Substitution - Missense
220	127	c.380C>G	p.S127C	COSM45483	1	Substitution - Missense
221	127	c.380C>T	p.S127F	COSM44226	13	Substitution - Missense
222	127	c.?	p.S127F	COSM1651680	3	Substitution - Missense
223	127	c.?	p.S127Y	COSM6024039	1	Substitution - Missense
224	128	c.383C>T	p.P128L	COSM45131	1	Substitution - Missense
225	130	c.388C>G	p.L130V	COSM11462	1	Substitution - Missense
226	130	c.388C>T	p.L130F	COSM11449	3	Substitution - Missense
227	130	c.389T>A	p.L130H	COSM46114	1	Substitution - Missense
228	130	c.389T>C	p.L130P	COSM45481	2	Substitution - Missense
229	130	c.?	p.L130F	COSM9973967	2	Substitution - Missense
230	131	c.392A>G	p.N131S	COSM44474	1	Substitution - Missense
231	131	c.392A>T	p.N131I	COSM44794	2	Substitution - Missense
232	132	c.394A>C	p.K132Q	COSM11224	1	Substitution - Missense
233	132	c.394A>G	p.K132E	COSM10813	5	Substitution - Missense
234	132	c.395A>C	p.K132T	COSM43912	2	Substitution - Missense
235	132	c.395A>G	p.K132R	COSM11582	2	Substitution - Missense
236	132	c.395A>T	p.K132M	COSM43592	1	Substitution - Missense
237	132	c.396G>C	p.K132N	COSM43963	4	Substitution - Missense
238	132	c.396G>T	p.K132N	COSM10991	2	Substitution - Missense
239	132	c.?	p.K132M	COSM7002263	1	Substitution - Missense
240	132	c.?	p.K132R	COSM166371	2	Substitution - Missense
241	133	c.398T>G	p.M133R	COSM43730	1	Substitution - Missense
242	133	c.?	p.M133V	COSM221570	1	Substitution - Missense
243	134	c.400T>G	p.F134V	COSM43941	1	Substitution - Missense
244	134	c.401T>C	p.F134S	COSM44506	1	Substitution - Missense
245	134	c.401T>G	p.F134C	COSM43949	2	Substitution - Missense
246	134	c.?	p.F134L	COSM1169422	1	Substitution - Missense
247	135	c.403T>A	p.C135S	COSM44910	1	Substitution - Missense
248	135	c.403T>G	p.C135G	COSM44829	1	Substitution - Missense
249	135	c.404G>A	p.C135Y	COSM10801	4	Substitution - Missense
250	135	c.405C>G	p.C135W	COSM44219	5	Substitution - Missense
251	135	c.?	p.C135G	COSM221571	1	Substitution - Missense
252	135	c.?	p.C135W	COSM4169704	1	Substitution - Missense
253	135	c.?	p.C135Y	COSM674226	1	Substitution - Missense
254	136	c.406C>G	p.Q136E	COSM43767	5	Substitution - Missense
255	138	c.412G>A	p.A138T	COSM44821	1	Substitution - Missense
256	138	c.412G>C	p.A138P	COSM11188	3	Substitution - Missense
257	138	c.413C>T	p.A138V	COSM43818	4	Substitution - Missense
258	138	c.?	p.A138P	COSM5546571	2	Substitution - Missense
259	138	c.?	p.A138V	COSM330609	1	Substitution - Missense
260	139	c.417G>C	p.K139N	COSM44101	2	Substitution - Missense
261	140	c.419C>T	p.T140I	COSM43742	4	Substitution - Missense
262	141	c.421T>C	p.C141R	COSM43901	1	Substitution - Missense
263	141	c.421T>G	p.C141G	COSM45794	1	Substitution - Missense
264	141	c.422G>A	p.C141Y	COSM43708	9	Substitution - Missense
265	141	c.422G>T	p.C141F	COSM44911	1	Substitution - Missense
266	141	c.423C>G	p.C141W	COSM44204	1	Substitution - Missense
267	142	c.425C>T	p.P142L	COSM43583	1	Substitution - Missense
268	143	c.427G>A	p.V143M	COSM43878	2	Substitution - Missense
269	145	c.433C>G	p.L145V	COSM45885	1	Substitution - Missense
270	145	c.434T>C	p.L145P	COSM43899	3	Substitution - Missense
271	146	c.436T>G	p.W146G	COSM44555	1	Substitution - Missense
272	151	c.451C>A	p.P151T	COSM43911	2	Substitution - Missense
273	151	c.451C>G	p.P151A	COSM44944	1	Substitution - Missense
274	151	c.451C>T	p.P151S	COSM10905	7	Substitution - Missense
275	151	c.452C>G	p.P151R	COSM44003	1	Substitution - Missense
276	151	c.452C>T	p.P151L	COSM44288	1	Substitution - Missense
277	151	c.?	p.P151S	COSM133656	2	Substitution - Missense
278	152	c.454C>A	p.P152T	COSM44561	3	Substitution - Missense
279	152	c.454C>G	p.P152A	COSM44788	1	Substitution - Missense
280	152	c.454C>T	p.P152S	COSM43582	1	Substitution - Missense
281	152	c.455C>A	p.P152Q	COSM44613	1	Substitution - Missense
282	152	c.455C>T	p.P152L	COSM10790	21	Substitution - Missense
283	152	c.?	p.P152L	COSM143793	1	Substitution - Missense
284	152	c.?	p.P152R	COSM1716294	1	Substitution - Missense
285	152	c.?	p.P152S	COSM4745862	1	Substitution - Missense
286	153	c.458C>T	p.P153L	COSM44367	1	Substitution - Missense
287	155	c.463A>C	p.T155P	COSM10912	1	Substitution - Missense
288	155	c.463A>G	p.T155A	COSM44303	1	Substitution - Missense
289	155	c.464C>A	p.T155N	COSM11218	7	Substitution - Missense
290	155	c.464C>T	p.T155I	COSM44033	1	Substitution - Missense
291	156	c.466C>A	p.R156S	COSM43744	2	Substitution - Missense
292	156	c.466C>T	p.R156C	COSM46124	1	Substitution - Missense
293	156	c.467G>A	p.R156H	COSM43739	1	Substitution - Missense
294	156	c.467G>C	p.R156P	COSM10760	1	Substitution - Missense
295	156	c.467G>T	p.R156L	COSM43548	1	Substitution - Missense
296	156	c.?	p.R156G	COSM4766148	1	Substitution - Missense
297	157	c.469G>T	p.V157F	COSM10670	9	Substitution - Missense
298	157	c.470T>A	p.V157D	COSM44329	1	Substitution - Missense
299	157	c.470T>G	p.V157G	COSM43903	3	Substitution - Missense
300	157	c.?	p.V157F	COSM144151	6	Substitution - Missense
301	158	c.472C>A	p.R158S	COSM3970360	2	Substitution - Missense
302	158	c.472C>G	p.R158G	COSM11087	8	Substitution - Missense
303	158	c.472C>T	p.R158C	COSM43848	2	Substitution - Missense
304	158	c.473G>A	p.R158H	COSM10690	21	Substitution - Missense
305	158	c.473G>C	p.R158P	COSM43615	3	Substitution - Missense
306	158	c.473G>T	p.R158L	COSM10714	3	Substitution - Missense
307	158	c.473_474delinsTT	p.R158L	COSM44974	1	Substitution - Missense
308	158	c.?	p.R158G	COSM1651671	1	Substitution - Missense
309	158	c.?	p.R158H	COSM330667	2	Substitution - Missense
310	159	c.475G>C	p.A159P	COSM43836	1	Substitution - Missense
311	159	c.476C>A	p.A159D	COSM11496	2	Substitution - Missense
312	159	c.476C>T	p.A159V	COSM11148	3	Substitution - Missense
313	161	c.481G>A	p.A161T	COSM10739	7	Substitution - Missense
314	161	c.481G>T	p.A161S	COSM43549	1	Substitution - Missense
315	161	c.482C>A	p.A161D	COSM11323	2	Substitution - Missense
316	161	c.482C>G	p.A161G	COSM46279	1	Substitution - Missense
317	161	c.482C>T	p.A161V	COSM43689	1	Substitution - Missense
318	161	c.?	p.A161T	COSM306126	7	Substitution - Missense
319	162	c.484A>G	p.I162V	COSM44413	1	Substitution - Missense
320	162	c.484A>T	p.I162F	COSM44320	1	Substitution - Missense
321	162	c.?	p.I162F	COSM4745852	1	Substitution - Missense
322	163	c.487T>A	p.Y163N	COSM44623	2	Substitution - Missense
323	163	c.487T>C	p.Y163H	COSM43846	2	Substitution - Missense
324	163	c.488A>G	p.Y163C	COSM10808	13	Substitution - Missense
325	163	c.?	p.Y163H	COSM5945683	1	Substitution - Missense
326	164	c.490A>G	p.K164E	COSM10762	2	Substitution - Missense
327	164	c.492G>T	p.K164N	COSM11369	1	Substitution - Missense
328	164	c.?	p.K164E	COSM1169394	1	Substitution - Missense
329	165	c.495G>C	p.Q165H	COSM4302127	1	Substitution - Missense
330	165	c.495G>T	p.Q165H	COSM44558	1	Substitution - Missense
331	165	c.?	p.Q165P	COSM4766149	1	Substitution - Missense
332	166	c.496_497delinsGG	p.S166G	COSM43932	1	Substitution - Missense
333	168	c.503A>G	p.H168R	COSM43545	3	Substitution - Missense
334	168	c.?	p.H168R	COSM4745864	1	Substitution - Missense
335	168	c.?	p.H168Y	COSM7002258	1	Substitution - Missense
336	170	c.509C>T	p.T170M	COSM44552	1	Substitution - Missense
337	171	c.512A>G	p.E171G	COSM44732	2	Substitution - Missense
338	172	c.514G>T	p.V172F	COSM44240	1	Substitution - Missense
339	172	c.515T>G	p.V172G	COSM45047	2	Substitution - Missense
340	172	c.?	p.V172F	COSM133680	1	Substitution - Missense
341	173	c.517G>A	p.V173M	COSM11084	5	Substitution - Missense
342	173	c.517G>C	p.V173L	COSM44057	1	Substitution - Missense
343	173	c.517G>T	p.V173L	COSM43559	7	Substitution - Missense
344	173	c.518T>C	p.V173A	COSM44327	7	Substitution - Missense
345	173	c.518T>G	p.V173G	COSM44383	1	Substitution - Missense
346	173	c.?	p.V173A	COSM7002265	1	Substitution - Missense
347	174	c.520A>T	p.R174W	COSM44782	1	Substitution - Missense
348	174	c.521G>A	p.R174K	COSM44524	1	Substitution - Missense
349	175	c.523C>A	p.R175S	COSM43931	1	Substitution - Missense
350	175	c.523C>G	p.R175G	COSM10870	2	Substitution - Missense
351	175	c.523C>T	p.R175C	COSM43680	2	Substitution - Missense
352	175	c.524G>A	p.R175H	COSM10648	123	Substitution - Missense
353	175	c.524G>C	p.R175P	COSM45416	1	Substitution - Missense
354	175	c.524G>T	p.R175L	COSM10718	1	Substitution - Missense
355	175	c.?	p.R175C	COSM4169629	1	Substitution - Missense
356	175	c.?	p.R175H	COSM144210	19	Substitution - Missense
357	175	c.?	p.R175S	COSM221568	1	Substitution - Missense
358	176	c.526T>C	p.C176R	COSM44948	3	Substitution - Missense
359	176	c.527G>A	p.C176Y	COSM10687	8	Substitution - Missense
360	176	c.527G>T	p.C176F	COSM10645	13	Substitution - Missense
361	177	c.529C>T	p.P177S	COSM10650	1	Substitution - Missense
362	177	c.530C>A	p.P177H	COSM45326	1	Substitution - Missense
363	177	c.530C>G	p.P177R	COSM10651	5	Substitution - Missense
364	177	c.530C>T	p.P177L	COSM44097	1	Substitution - Missense
365	178	c.533A>C	p.H178P	COSM44215	4	Substitution - Missense
366	178	c.534C>A	p.H178Q	COSM11998	2	Substitution - Missense
367	179	c.535C>A	p.H179N	COSM44151	1	Substitution - Missense
368	179	c.535C>G	p.H179D	COSM44776	4	Substitution - Missense
369	179	c.535C>T	p.H179Y	COSM10768	8	Substitution - Missense
370	179	c.536A>C	p.H179P	COSM44218	1	Substitution - Missense
371	179	c.536A>G	p.H179R	COSM10889	32	Substitution - Missense
372	179	c.536A>T	p.H179L	COSM43635	1	Substitution - Missense
373	179	c.537T>A	p.H179Q	COSM44214	1	Substitution - Missense
374	179	c.537T>G	p.H179Q	COSM11249	2	Substitution - Missense
375	179	c.?	p.H179D	COSM7002262	1	Substitution - Missense
376	179	c.?	p.H179Y	COSM133679	1	Substitution - Missense
377	180	c.?	p.E180K	COSM330664	1	Substitution - Missense
378	181	c.541C>T	p.R181C	COSM11090	5	Substitution - Missense
379	181	c.542G>A	p.R181H	COSM10738	4	Substitution - Missense
380	181	c.542G>C	p.R181P	COSM45046	3	Substitution - Missense
381	183	c.547T>C	p.S183P	COSM44343	1	Substitution - Missense
382	184	c.550G>T	p.D184Y	COSM44202	1	Substitution - Missense
383	186	c.557A>G	p.D186G	COSM46287	1	Substitution - Missense
384	187	c.?	p.G187S	COSM219079	1	Substitution - Missense
385	188	c.562C>A	p.L188M	COSM3727816	1	Substitution - Missense
386	190	c.568C>T	p.P190S	COSM44682	4	Substitution - Missense
387	190	c.569C>G	p.P190R	COSM44004	3	Substitution - Missense
388	190	c.569C>T	p.P190L	COSM43657	6	Substitution - Missense
389	190	c.?	p.P190L	COSM133661	3	Substitution - Missense
390	193	c.577C>A	p.H193N	COSM43935	2	Substitution - Missense
391	193	c.577C>G	p.H193D	COSM44002	1	Substitution - Missense
392	193	c.577C>T	p.H193Y	COSM10672	10	Substitution - Missense
393	193	c.578A>C	p.H193P	COSM43833	3	Substitution - Missense
394	193	c.578A>G	p.H193R	COSM10742	17	Substitution - Missense
395	193	c.578A>T	p.H193L	COSM11066	4	Substitution - Missense
396	193	c.?	p.H193L	COSM330673	1	Substitution - Missense
397	193	c.?	p.H193R	COSM1716523	1	Substitution - Missense
398	193	c.?	p.H193Y	COSM7002267	1	Substitution - Missense
399	194	c.581T>G	p.L194R	COSM44571	2	Substitution - Missense
400	194	c.?	p.L194R	COSM4172004	2	Substitution - Missense
401	195	c.583A>T	p.I195F	COSM44633	2	Substitution - Missense
402	195	c.584T>A	p.I195N	COSM44877	2	Substitution - Missense
403	195	c.584T>C	p.I195T	COSM11089	11	Substitution - Missense
404	195	c.?	p.I195F	COSM1666654	1	Substitution - Missense
405	196	c.587G>A	p.R196Q	COSM44599	1	Substitution - Missense
406	196	c.587G>C	p.R196P	COSM43814	2	Substitution - Missense
407	197	c.590T>A	p.V197E	COSM44424	1	Substitution - Missense
408	199	c.595G>A	p.G199R	COSM43749	3	Substitution - Missense
409	199	c.596G>A	p.G199E	COSM43989	1	Substitution - Missense
410	199	c.596G>T	p.G199V	COSM44140	2	Substitution - Missense
411	199	c.?	p.G199E	COSM5985110	1	Substitution - Missense
412	200	c.599A>C	p.N200T	COSM45331	1	Substitution - Missense
413	203	c.607G>A	p.V203M	COSM43599	1	Substitution - Missense
414	205	c.613T>C	p.Y205H	COSM43642	1	Substitution - Missense
415	205	c.613T>G	p.Y205D	COSM43844	2	Substitution - Missense
416	205	c.614A>C	p.Y205S	COSM44169	4	Substitution - Missense
417	205	c.614A>G	p.Y205C	COSM43947	6	Substitution - Missense
418	205	c.?	p.Y205C	COSM330677	1	Substitution - Missense
419	205	c.?	p.Y205H	COSM146252	1	Substitution - Missense
420	208	c.623A>G	p.D208G	COSM45796	1	Substitution - Missense
421	208	c.623A>T	p.D208V	COSM44249	2	Substitution - Missense
422	208	c.624C>A	p.D208E	COSM45707	1	Substitution - Missense
423	208	c.?	p.D208Y	COSM4745859	1	Substitution - Missense
424	210	c.628A>G	p.N210D	COSM43933	1	Substitution - Missense
425	211	c.632C>T	p.T211I	COSM43939	6	Substitution - Missense
426	213	c.637C>G	p.R213G	COSM44102	1	Substitution - Missense
427	213	c.638G>A	p.R213Q	COSM10735	5	Substitution - Missense
428	213	c.638G>T	p.R213L	COSM43650	2	Substitution - Missense
429	213	c.?	p.R213Q	COSM146253	3	Substitution - Missense
430	213	c.?	p.R213W	COSM4745857	1	Substitution - Missense
431	214	c.640C>G	p.H214D	COSM45115	1	Substitution - Missense
432	214	c.641A>G	p.H214R	COSM43687	11	Substitution - Missense
433	214	c.?	p.H214R	COSM1169410	1	Substitution - Missense
434	215	c.643A>G	p.S215G	COSM43951	5	Substitution - Missense
435	215	c.644G>A	p.S215N	COSM44093	1	Substitution - Missense
436	215	c.644G>C	p.S215T	COSM44175	1	Substitution - Missense
437	215	c.644G>T	p.S215I	COSM11450	2	Substitution - Missense
438	215	c.?	p.S215R	COSM3734734	1	Substitution - Missense
439	216	c.646G>A	p.V216M	COSM10667	11	Substitution - Missense
440	216	c.646G>T	p.V216L	COSM11210	1	Substitution - Missense
441	216	c.?	p.V216M	COSM143796	1	Substitution - Missense
442	217	c.649G>T	p.V217L	COSM44334	1	Substitution - Missense
443	217	c.?	p.V217G	COSM9989106	1	Substitution - Missense
444	218	c.653T>G	p.V218G	COSM44198	2	Substitution - Missense
445	219	c.656C>G	p.P219R	COSM44053	1	Substitution - Missense
446	220	c.658T>C	p.Y220H	COSM44637	1	Substitution - Missense
447	220	c.658T>G	p.Y220D	COSM11847	1	Substitution - Missense
448	220	c.659A>C	p.Y220S	COSM43850	1	Substitution - Missense
449	220	c.659A>G	p.Y220C	COSM10758	39	Substitution - Missense
450	220	c.?	p.Y220C	COSM96438	4	Substitution - Missense
451	220	c.?	p.Y220S	COSM1577275	2	Substitution - Missense
452	222	c.665C>T	p.P222L	COSM44606	1	Substitution - Missense
453	224	c.671A>G	p.E224G	COSM44014	1	Substitution - Missense
454	224	c.672G>C	p.E224D	COSM44945	1	Substitution - Missense
455	224	c.672G>T	p.E224D	COSM11451	1	Substitution - Missense
456	227	c.680C>T	p.S227F	COSM43920	1	Substitution - Missense
457	228	c.682G>T	p.D228Y	COSM45786	1	Substitution - Missense
458	230	c.688A>C	p.T230P	COSM44271	1	Substitution - Missense
459	230	c.689C>T	p.T230I	COSM43868	2	Substitution - Missense
460	232	c.695T>G	p.I232S	COSM45045	2	Substitution - Missense
461	234	c.700T>A	p.Y234N	COSM43956	1	Substitution - Missense
462	234	c.700T>C	p.Y234H	COSM11152	2	Substitution - Missense
463	234	c.700T>G	p.Y234D	COSM43768	1	Substitution - Missense
464	234	c.701A>G	p.Y234C	COSM10725	13	Substitution - Missense
465	234	c.?	p.Y234H	COSM4766150	1	Substitution - Missense
466	235	c.703A>G	p.N235D	COSM11542	1	Substitution - Missense
467	235	c.704A>G	p.N235S	COSM43616	3	Substitution - Missense
468	236	c.706T>A	p.Y236N	COSM43826	2	Substitution - Missense
469	236	c.706T>G	p.Y236D	COSM43602	1	Substitution - Missense
470	236	c.707A>C	p.Y236S	COSM44693	1	Substitution - Missense
471	236	c.707A>G	p.Y236C	COSM10731	5	Substitution - Missense
472	236	c.?	p.Y236N	COSM1169561	1	Substitution - Missense
473	237	c.709A>G	p.M237V	COSM44525	2	Substitution - Missense
474	237	c.711G>A	p.M237I	COSM10834	14	Substitution - Missense
475	237	c.711G>T	p.M237I	COSM11063	3	Substitution - Missense
476	237	c.?	p.M237I	COSM46274	5	Substitution - Missense
477	238	c.713G>A	p.C238Y	COSM11059	11	Substitution - Missense
478	238	c.713G>C	p.C238S	COSM44653	3	Substitution - Missense
479	238	c.713G>T	p.C238F	COSM43778	3	Substitution - Missense
480	238	c.?	p.C238Y	COSM166370	2	Substitution - Missense
481	239	c.715A>G	p.N239D	COSM10777	2	Substitution - Missense
482	239	c.716A>G	p.N239S	COSM44094	1	Substitution - Missense
483	239	c.717C>G	p.N239K	COSM44510	1	Substitution - Missense
484	239	c.?	p.N239T	COSM4603632	1	Substitution - Missense
485	240	c.718A>G	p.S240G	COSM43973	3	Substitution - Missense
486	240	c.719G>C	p.S240T	COSM44964	1	Substitution - Missense
487	241	c.721T>C	p.S241P	COSM44578	1	Substitution - Missense
488	241	c.721T>G	p.S241A	COSM44224	2	Substitution - Missense
489	241	c.722C>A	p.S241Y	COSM10935	3	Substitution - Missense
490	241	c.722C>T	p.S241F	COSM10812	11	Substitution - Missense
491	241	c.?	p.S241F	COSM329738	4	Substitution - Missense
492	241	c.?	p.S241Y	COSM1717459	1	Substitution - Missense
493	242	c.724T>A	p.C242S	COSM44935	1	Substitution - Missense
494	242	c.724T>C	p.C242R	COSM11738	1	Substitution - Missense
495	242	c.725G>A	p.C242Y	COSM10646	6	Substitution - Missense
496	242	c.725G>C	p.C242S	COSM11133	2	Substitution - Missense
497	242	c.725G>T	p.C242F	COSM10810	3	Substitution - Missense
498	242	c.?	p.C242S	COSM330665	1	Substitution - Missense
499	242	c.?	p.C242Y	COSM330672	2	Substitution - Missense
500	243	c.727A>C	p.M243L	COSM43765	1	Substitution - Missense
501	244	c.730G>A	p.G244S	COSM10941	6	Substitution - Missense
502	244	c.730G>C	p.G244R	COSM44221	1	Substitution - Missense
503	244	c.731G>A	p.G244D	COSM10883	5	Substitution - Missense
504	244	c.731G>T	p.G244V	COSM43652	1	Substitution - Missense
505	244	c.?	p.G244S	COSM330669	2	Substitution - Missense
506	245	c.733G>A	p.G245S	COSM6932	52	Substitution - Missense
507	245	c.733G>C	p.G245R	COSM10957	1	Substitution - Missense
508	245	c.733G>T	p.G245C	COSM11081	1	Substitution - Missense
509	245	c.734G>A	p.G245D	COSM43606	5	Substitution - Missense
510	245	c.734G>T	p.G245V	COSM11196	5	Substitution - Missense
511	245	c.?	p.G245D	COSM143788	5	Substitution - Missense
512	245	c.?	p.G245S	COSM145022	14	Substitution - Missense
513	245	c.?	p.G245V	COSM1162159	1	Substitution - Missense
514	246	c.736A>G	p.M246V	COSM43555	4	Substitution - Missense
515	246	c.737T>C	p.M246T	COSM11355	1	Substitution - Missense
516	246	c.737T>G	p.M246R	COSM11376	3	Substitution - Missense
517	246	c.738G>A	p.M246I	COSM44310	2	Substitution - Missense
518	246	c.?	p.M246I	COSM1732649	2	Substitution - Missense
519	246	c.?	p.M246T	COSM330615	1	Substitution - Missense
520	248	c.742C>G	p.R248G	COSM11564	1	Substitution - Missense
521	248	c.742C>T	p.R248W	COSM10656	63	Substitution - Missense
522	248	c.743G>A	p.R248Q	COSM10662	77	Substitution - Missense
523	248	c.743G>T	p.R248L	COSM6549	1	Substitution - Missense
524	248	c.?	p.R248Q	COSM87196	12	Substitution - Missense
525	248	c.?	p.R248W	COSM144150	7	Substitution - Missense
526	249	c.745A>G	p.R249G	COSM10668	5	Substitution - Missense
527	249	c.746G>C	p.R249T	COSM43665	4	Substitution - Missense
528	249	c.746G>T	p.R249M	COSM43871	1	Substitution - Missense
529	249	c.747G>C	p.R249S	COSM10785	1	Substitution - Missense
530	249	c.747G>T	p.R249S	COSM10817	4	Substitution - Missense
531	249	c.?	p.R249S	COSM133090	1	Substitution - Missense
532	250	c.749C>G	p.P250R	COSM8257737	1	Substitution - Missense
533	250	c.749C>T	p.P250L	COSM10771	6	Substitution - Missense
534	251	c.751A>C	p.I251L	COSM10931	1	Substitution - Missense
535	251	c.752T>G	p.I251S	COSM43829	1	Substitution - Missense
536	252	c.755T>A	p.L252H	COSM45091	1	Substitution - Missense
537	252	c.755T>C	p.L252P	COSM44769	2	Substitution - Missense
538	252	c.?	p.L252P	COSM7002266	1	Substitution - Missense
539	254	c.760A>C	p.I254L	COSM1579892	1	Substitution - Missense
540	254	c.761T>C	p.I254T	COSM44058	1	Substitution - Missense
541	254	c.761T>G	p.I254S	COSM45035	4	Substitution - Missense
542	255	c.763A>T	p.I255F	COSM43651	2	Substitution - Missense
543	255	c.764T>A	p.I255N	COSM11244	1	Substitution - Missense
544	255	c.764T>G	p.I255S	COSM10788	2	Substitution - Missense
545	256	c.767C>A	p.T256K	COSM44429	2	Substitution - Missense
546	256	c.?	p.T256K	COSM7002323	2	Substitution - Missense
547	257	c.769C>G	p.L257V	COSM43699	2	Substitution - Missense
548	257	c.770T>A	p.L257Q	COSM43530	1	Substitution - Missense
549	258	c.772G>A	p.E258K	COSM10988	3	Substitution - Missense
550	258	c.772G>C	p.E258Q	COSM10751	1	Substitution - Missense
551	258	c.773A>C	p.E258A	COSM44719	1	Substitution - Missense
552	258	c.773A>G	p.E258G	COSM44168	1	Substitution - Missense
553	258	c.773A>T	p.E258V	COSM44450	5	Substitution - Missense
554	258	c.?	p.E258K	COSM1732658	1	Substitution - Missense
555	258	c.?	p.E258Q	COSM5945719	1	Substitution - Missense
556	259	c.775G>C	p.D259H	COSM46185	1	Substitution - Missense
557	259	c.776A>T	p.D259V	COSM43724	1	Substitution - Missense
558	262	c.785G>T	p.G262V	COSM11198	2	Substitution - Missense
559	263	c.?	p.N263K	COSM7002264	1	Substitution - Missense
560	265	c.794T>C	p.L265P	COSM11011	1	Substitution - Missense
561	265	c.794T>G	p.L265R	COSM44092	1	Substitution - Missense
562	266	c.796G>A	p.G266R	COSM10794	13	Substitution - Missense
563	266	c.796G>C	p.G266R	COSM11205	2	Substitution - Missense
564	266	c.797G>A	p.G266E	COSM10867	5	Substitution - Missense
565	266	c.797G>T	p.G266V	COSM10958	2	Substitution - Missense
566	266	c.?	p.G266E	COSM330616	1	Substitution - Missense
567	266	c.?	p.G266R	COSM6908524	1	Substitution - Missense
568	267	c.799C>T	p.R267W	COSM11183	15	Substitution - Missense
569	267	c.800G>A	p.R267Q	COSM43923	4	Substitution - Missense
570	267	c.800G>C	p.R267P	COSM11392	2	Substitution - Missense
571	267	c.?	p.R267W	COSM1169538	1	Substitution - Missense
572	269	c.806G>T	p.S269I	COSM4302129	2	Substitution - Missense
573	270	c.808T>C	p.F270L	COSM44262	1	Substitution - Missense
574	270	c.809T>C	p.F270S	COSM11305	3	Substitution - Missense
575	271	c.811G>A	p.E271K	COSM10719	2	Substitution - Missense
576	271	c.812A>G	p.E271G	COSM43879	2	Substitution - Missense
577	272	c.814G>A	p.V272M	COSM10891	9	Substitution - Missense
578	272	c.814G>T	p.V272L	COSM10859	3	Substitution - Missense
579	272	c.815T>C	p.V272A	COSM44294	1	Substitution - Missense
580	272	c.?	p.V272L	COSM133678	3	Substitution - Missense
581	272	c.?	p.V272M	COSM166369	1	Substitution - Missense
582	273	c.817C>G	p.R273G	COSM43843	1	Substitution - Missense
583	273	c.817C>T	p.R273C	COSM10659	277	Substitution - Missense
584	273	c.817_818delinsTA	p.R273Y	COSM6906023	1	Substitution - Missense
585	273	c.818G>A	p.R273H	COSM10660	87	Substitution - Missense
586	273	c.818G>C	p.R273P	COSM43896	3	Substitution - Missense
587	273	c.818G>T	p.R273L	COSM10779	6	Substitution - Missense
588	273	c.?	p.R273C	COSM144162	43	Substitution - Missense
589	273	c.?	p.R273H	COSM133677	20	Substitution - Missense
590	273	c.?	p.R273L	COSM144153	2	Substitution - Missense
591	274	c.820G>A	p.V274I	COSM43667	1	Substitution - Missense
592	274	c.820G>T	p.V274F	COSM10769	1	Substitution - Missense
593	274	c.821T>C	p.V274A	COSM44393	2	Substitution - Missense
594	274	c.821T>G	p.V274G	COSM43945	1	Substitution - Missense
595	275	c.823T>C	p.C275R	COSM43902	1	Substitution - Missense
596	275	c.823T>G	p.C275G	COSM11501	3	Substitution - Missense
597	275	c.824G>A	p.C275Y	COSM10893	8	Substitution - Missense
598	275	c.824G>T	p.C275F	COSM10701	9	Substitution - Missense
599	275	c.825T>G	p.C275W	COSM43823	1	Substitution - Missense
600	275	c.?	p.C275R	COSM329747	2	Substitution - Missense
601	275	c.?	p.C275Y	COSM330617	1	Substitution - Missense
602	276	c.827C>A	p.A276D	COSM45268	4	Substitution - Missense
603	276	c.827C>G	p.A276G	COSM45695	1	Substitution - Missense
604	276	c.827C>T	p.A276V	COSM10756	2	Substitution - Missense
605	276	c.?	p.A276G	COSM1732655	1	Substitution - Missense
606	277	c.829T>C	p.C277R	COSM45871	1	Substitution - Missense
607	277	c.830G>A	p.C277Y	COSM43737	1	Substitution - Missense
608	277	c.830G>T	p.C277F	COSM10749	4	Substitution - Missense
609	277	c.831T>G	p.C277W	COSM45299	1	Substitution - Missense
610	278	c.832C>A	p.P278T	COSM43697	2	Substitution - Missense
611	278	c.832C>G	p.P278A	COSM10814	1	Substitution - Missense
612	278	c.832C>T	p.P278S	COSM10939	5	Substitution - Missense
613	278	c.833C>A	p.P278H	COSM43755	3	Substitution - Missense
614	278	c.833C>G	p.P278R	COSM10887	1	Substitution - Missense
615	278	c.833C>T	p.P278L	COSM10863	6	Substitution - Missense
616	278	c.?	p.P278L	COSM5352238	1	Substitution - Missense
617	278	c.?	p.P278S	COSM330674	2	Substitution - Missense
618	279	c.836G>A	p.G279E	COSM43714	8	Substitution - Missense
619	279	c.?	p.G279E	COSM4603630	1	Substitution - Missense
620	280	c.838A>G	p.R280G	COSM11123	4	Substitution - Missense
621	280	c.839G>A	p.R280K	COSM10728	4	Substitution - Missense
622	280	c.839G>C	p.R280T	COSM10724	3	Substitution - Missense
623	280	c.839G>T	p.R280I	COSM11287	1	Substitution - Missense
624	280	c.840A>T	p.R280S	COSM44171	1	Substitution - Missense
625	280	c.?	p.R280G	COSM1666590	2	Substitution - Missense
626	280	c.?	p.R280I	COSM6024595	1	Substitution - Missense
627	280	c.?	p.R280T	COSM166372	2	Substitution - Missense
628	281	c.841G>A	p.D281N	COSM43596	4	Substitution - Missense
629	281	c.841G>C	p.D281H	COSM10943	9	Substitution - Missense
630	281	c.842A>C	p.D281A	COSM11665	2	Substitution - Missense
631	281	c.842A>G	p.D281G	COSM11232	1	Substitution - Missense
632	281	c.842A>T	p.D281V	COSM45729	1	Substitution - Missense
633	281	c.843C>G	p.D281E	COSM43837	3	Substitution - Missense
634	281	c.843_844delinsGT	p.D281_R282delinsEW	COSM45816	1	Substitution - Missense
635	281	c.?	p.D281E	COSM133657	1	Substitution - Missense
636	281	c.?	p.D281H	COSM7338405	1	Substitution - Missense
637	282	c.844C>G	p.R282G	COSM10992	1	Substitution - Missense
638	282	c.844C>T	p.R282W	COSM10704	60	Substitution - Missense
639	282	c.845G>A	p.R282Q	COSM44338	1	Substitution - Missense
640	282	c.?	p.R282W	COSM96436	7	Substitution - Missense
641	283	c.847C>T	p.R283C	COSM10911	2	Substitution - Missense
642	283	c.848G>C	p.R283P	COSM10743	1	Substitution - Missense
643	283	c.?	p.R283C	COSM87043	2	Substitution - Missense
644	283	c.?	p.R283H	COSM6834791	1	Substitution - Missense
645	284	c.850A>T	p.T284S	COSM4302128	1	Substitution - Missense
646	285	c.853G>A	p.E285K	COSM10722	4	Substitution - Missense
647	285	c.854A>T	p.E285V	COSM44227	1	Substitution - Missense
648	286	c.856G>A	p.E286K	COSM10726	5	Substitution - Missense
649	286	c.856G>C	p.E286Q	COSM44250	1	Substitution - Missense
650	286	c.857A>G	p.E286G	COSM43565	2	Substitution - Missense
651	286	c.857A>T	p.E286V	COSM43936	1	Substitution - Missense
652	286	c.?	p.E286G	COSM4745865	1	Substitution - Missense
653	289	c.866T>G	p.L289R	COSM44344	1	Substitution - Missense
654	290	c.869G>A	p.R290H	COSM44017	1	Substitution - Missense
655	291	c.872A>G	p.K291R	COSM43747	1	Substitution - Missense
656	293	c.877G>T	p.G293W	COSM46261	1	Substitution - Missense
657	294	c.881A>G	p.E294G	COSM43746	1	Substitution - Missense
658	298	c.894G>C	p.E298D	COSM4589938	1	Substitution - Missense
659	305	c.914A>G	p.K305R	COSM43743	2	Substitution - Missense
660	307	c.920C>T	p.A307V	COSM35846	2	Substitution - Missense
661	313	c.938G>A	p.S313N	COSM44557	1	Substitution - Missense
662	324	c.?	p.D324N	COSM9975897	1	Substitution - Missense
663	329	c.?	p.T329I	COSM5945722	1	Substitution - Missense
664	331	c.993G>T	p.Q331H	COSM96339	1	Substitution - Missense
665	335	c.?	p.R335C	COSM5945724	1	Substitution - Missense
666	336	c.?	p.E336K	COSM6849515	1	Substitution - Missense
667	337	c.1009C>T	p.R337C	COSM11071	9	Substitution - Missense
668	337	c.?	p.R337C	COSM6024625	1	Substitution - Missense
669	342	c.1025G>C	p.R342P	COSM45276	3	Substitution - Missense
670	347	c.1039G>A	p.A347T	COSM45717	2	Substitution - Missense
671	347	c.1040C>G	p.A347G	COSM46323	1	Substitution - Missense
672	347	c.?	p.A347T	COSM10134010	1	Substitution - Missense
673	364	c.1090G>A	p.A364T	COSM46361	1	Substitution - Missense
674	373	c.1118A>G	p.K373R	COSM3727817	1	Substitution - Missense
675	375	c.1123C>A	p.Q375K	COSM3403253	1	Substitution - Missense
676	379	c.1136G>A	p.R379H	COSM44189	2	Substitution - Missense
677	392	c.1174T>C	p.S392P	COSM6950049	1	Substitution - Missense
678	5	c.13C>T	p.Q5*	COSM44191	1	Substitution - Nonsense
679	23	c.68G>A	p.W23*	COSM6916925	1	Substitution - Nonsense
680	53	c.158G>A	p.W53*	COSM44760	3	Substitution - Nonsense
681	53	c.159G>A	p.W53*	COSM44488	2	Substitution - Nonsense
682	53	c.?	p.W53*	COSM329729	1	Substitution - Nonsense
683	56	c.166G>T	p.E56*	COSM12168	1	Substitution - Nonsense
684	68	c.?	p.E68*	COSM4603637	1	Substitution - Nonsense
685	91	c.272G>A	p.W91*	COSM44192	3	Substitution - Nonsense
686	91	c.?	p.W91*	COSM4169625	1	Substitution - Nonsense
687	94	c.281C>G	p.S94*	COSM45653	1	Substitution - Nonsense
688	100	c.298C>T	p.Q100*	COSM44032	1	Substitution - Nonsense
689	136	c.406C>T	p.Q136*	COSM11166	6	Substitution - Nonsense
690	141	c.?	p.C141*	COSM221573	1	Substitution - Nonsense
691	144	c.430C>T	p.Q144*	COSM11245	1	Substitution - Nonsense
692	146	c.437G>A	p.W146*	COSM43609	6	Substitution - Nonsense
693	146	c.438G>A	p.W146*	COSM10727	5	Substitution - Nonsense
694	164	c.490A>T	p.K164*	COSM10750	1	Substitution - Nonsense
695	167	c.499C>T	p.Q167*	COSM11333	1	Substitution - Nonsense
696	167	c.?	p.Q167*	COSM146254	1	Substitution - Nonsense
697	176	c.?	p.C176*	COSM4745860	1	Substitution - Nonsense
698	183	c.548C>G	p.S183*	COSM10706	4	Substitution - Nonsense
699	192	c.574C>T	p.Q192*	COSM10733	2	Substitution - Nonsense
700	196	c.586C>T	p.R196*	COSM10705	28	Substitution - Nonsense
701	196	c.?	p.R196*	COSM143787	2	Substitution - Nonsense
702	213	c.637C>T	p.R213*	COSM10654	23	Substitution - Nonsense
703	213	c.?	p.R213*	COSM166368	6	Substitution - Nonsense
704	221	c.661G>T	p.E221*	COSM44817	2	Substitution - Nonsense
705	234	c.702C>A	p.Y234*	COSM45114	2	Substitution - Nonsense
706	238	c.714T>A	p.C238*	COSM45677	1	Substitution - Nonsense
707	271	c.811G>T	p.E271*	COSM43750	1	Substitution - Nonsense
708	273	c.?	p.R273*	COSM144152	1	Substitution - Nonsense
709	286	c.856G>T	p.E286*	COSM43919	1	Substitution - Nonsense
710	298	c.?	p.E298*	COSM133654	1	Substitution - Nonsense
711	306	c.916C>T	p.R306*	COSM10663	21	Substitution - Nonsense
712	306	c.?	p.R306*	COSM145026	6	Substitution - Nonsense
713	317	c.949C>T	p.Q317*	COSM10786	3	Substitution - Nonsense
714	317	c.?	p.Q317*	COSM1169548	1	Substitution - Nonsense
715	320	c.958A>T	p.K320*	COSM44335	2	Substitution - Nonsense
716	327	c.981T>G	p.Y327*	COSM44823	1	Substitution - Nonsense
717	331	c.991C>T	p.Q331*	COSM11354	1	Substitution - Nonsense
718	336	c.1006G>T	p.E336*	COSM11291	2	Substitution - Nonsense
719	339	c.1015G>T	p.E339*	COSM11286	4	Substitution - Nonsense
720	342	c.1024C>T	p.R342*	COSM11073	22	Substitution - Nonsense
721	342	c.?	p.R342*	COSM306146	10	Substitution - Nonsense
722	348	c.1043T>A	p.L348*	COSM46015	1	Substitution - Nonsense
723	349	c.1045G>T	p.E349*	COSM10770	1	Substitution - Nonsense
724	191	c.?	p.?	COSM44855	1	Unknown
725	-	c.75-1G>A	p.?	COSM6983171	1	Unknown
726	-	c.96+1G>A	p.?	COSM44435	2	Unknown
727	-	c.96+1G>T	p.?	COSM307275	4	Unknown
728	-	c.97-11_103del	p.?	COSM6969464	1	Unknown
729	-	c.97-1G>A	p.?	COSM43759	1	Unknown
730	-	c.97-1G>C	p.?	COSM29761	1	Unknown
731	-	c.97-1G>T	p.?	COSM1610880	1	Unknown
732	-	c.97-2A>G	p.?	COSM39405	2	Unknown
733	-	c.375+1G>A	p.?	COSM45304	2	Unknown
734	-	c.375+1G>C	p.?	COSM45910	1	Unknown
735	-	c.375+6T>G	p.?	COSM4969482	1	Unknown
736	-	c.376-1G>A	p.?	COSM6900	5	Unknown
737	-	c.376-2A>C	p.?	COSM46049	1	Unknown
738	-	c.376-2A>G	p.?	COSM45672	1	Unknown
739	-	c.376-2del	p.?	COSM45658	1	Unknown
740	-	c.548_559+2del	p.?	COSM6905819	1	Unknown
741	-	c.559+13_559+14del	p.?	COSM44193	1	Unknown
742	-	c.559+1G>A	p.?	COSM6901	3	Unknown
743	-	c.560-1G>A	p.?	COSM43753	5	Unknown
744	-	c.560-22A>G	p.?	COSM45665	1	Unknown
745	-	c.560-2A>G	p.?	COSM18657	1	Unknown
746	-	c.560-3T>G	p.?	COSM46059	1	Unknown
747	-	c.672+1G>A	p.?	COSM6906	2	Unknown
748	-	c.672+2T>C	p.?	COSM45517	1	Unknown
749	-	c.673-1G>A	p.?	COSM43751	2	Unknown
750	-	c.673-1G>C	p.?	COSM45675	1	Unknown
751	-	c.673-2A>G	p.?	COSM6908	2	Unknown
752	-	c.673-41_673-33delinsCAGAGCCCA	p.?	COSM46260	1	Unknown
753	-	c.782+42C>T	p.?	COSM44039	1	Unknown
754	-	c.783-1G>T	p.?	COSM6913	1	Unknown
755	-	c.783-1_789del	p.?	COSM9114976	1	Unknown
756	-	c.783-2A>C	p.?	COSM13744	2	Unknown
757	-	c.919+1G>A	p.?	COSM44143	1	Unknown
758	-	c.919+1G>C	p.?	COSM13585	2	Unknown
759	-	c.919+1G>T	p.?	COSM13584	2	Unknown
760	-	c.919+6C>T	p.?	COSM44195	1	Unknown
761	-	c.920-1G>A	p.?	COSM6917	2	Unknown
762	-	c.920-2A>G	p.?	COSM33650	2	Unknown
763	-	c.987_993+6del	p.?	COSM4970898	1	Unknown
764	-	c.993+291G>A	p.?	COSM5977671	1	Unknown
765	-	c.993+2T>C	p.?	COSM45552	1	Unknown
766	-	c.993+982G>C	p.?	COSN6549382	1	Unknown
767	-	c.994-1G>A	p.?	COSM69404	2	Unknown
768	-	c.994-1G>C	p.?	COSM13745	1	Unknown
769	-	c.994-2A>C	p.?	COSM3970337	1	Unknown
770	-	c.994-2A>G	p.?	COSM87027	1	Unknown
771	-	c.*387C>A	p.?	COSN20093202	1	Unknown
772	-	c.?	p.?	COSM43617	385	Unknown
773	1	c.1_*del	p.0	COSM18654	4	Whole gene deletion
VEGFA	Position	CDS Mutation	AA Mutation	Legacy Mutation ID	Count	Type
1	97	c.291G>A	p.E97=	COSM9202275	1	Substitution - coding silent
2	265	c.795C>T	p.G265=	COSM9209991	3	Substitution - coding silent
3	239	c.715G>A	p.V239M	COSM6928938	1	Substitution - Missense
4	257	c.770G>A	p.C257Y	COSM6974477	1	Substitution - Missense
5	351	c.1052G>A	p.R351H	COSM296967	1	Substitution - Missense
6	323	c.969T>A	p.C323*	COSM7482303	1	Substitution - Nonsense
7	361	c.1081G>T	p.E361*	COSM8259731	1	Substitution - Nonsense
8	-	c.-363T>C	p.?	COSN6590948	1	Unknown
9	-	c.855+1G>A	p.?	COSM9209993	3	Unknown
10	-	c.963-1214C>T	p.?	COSM248279	1	Unknown
11	-	c.*60A>T	p.?	COSM3411133	1	Unknown
12	-	c.*78C>T	p.?	COSN30102404	1	Unknown
CTH	Position	CDS Mutation	AA Mutation	Legacy Mutation ID	Count	Type
1	62	c.185G>T	p.R62L	COSM3400990	1	Substitution - Missense
2	332	c.995G>T	p.R332I	COSM7473216	1	Substitution - Missense
3	-	c.-144C>G	p.?	COSN30101590	2	Unknown
4	-	c.*140T>A	p.?	COSN30104531	1	Unknown
